# Multidomain interaction governs the filamentous assembly of the dominant-negative DNMT3A R882H mutant

**DOI:** 10.1073/pnas.2609226123

**Published:** 2026-08-25

**Authors:** Jianbin Chen, Jiuwei Lu, Zhixu Long, Sol Yoon, Megan Fukunaga, Jikui Song

**Affiliations:** ^a^ Department of Biochemistry, University of California, Riverside, CA 92521

**Keywords:** DNMT3A, DNMT3A R882H, polymerization, DNA methylation

## Abstract

DNA methyltransferase DNMT3A-mediated de novo DNA methylation is important for proper regulation of gene expression and genomic stability in development. The DNMT3A R882H (DNMT3A^R882H^) mutation, a hot-spot mutation in acute myeloid leukemia and developmental disorders, exerts a dominant-negative effect in DNMT3A-mediated DNA methylation through promoting high-order protein assembly. However, due to the lack of structural knowledge on DNMT3A homo-oligomers, the mechanism behind wild-type DNMT3A (DNMT3A^WT^) and DNMT3A^R882H^ polymerization remains unclear. Here, we report the single-particle cryo-EM structures of homo-oligomeric DNMT3A^WT^ and filamentous DNMT3A^R882H^, revealing the role of the regulatory Pro-Trp-Trp-Pro (PWWP) and ATRX-DNMT3-DNMT3L (ADD) domains of DNMT3A in their dynamic assembly. While the oligomeric assembly of DNMT3A is mainly driven by the well-characterized oligomer interfaces in the methyltransferase domain, the autoinhibitory interaction of the PWWP and ADD domains in DNMT3A places them in a position for intermolecular contact, thereby contributing to the filamentous assembly of DNMT3A^R882H^. Disrupting the autoinhibitory interaction facilitates the transition of DNMT3A^R882H^ polymer toward the low-order oligomeric assembly, reinforcing the aggregation-attenuation effect of the previously characterized oligomer-interface mutation R676K. Together, this study uncovers a multidomain cooperated assembly mechanism for DNMT3A, with important implication in development of effective therapeutic strategies against DNMT3A^R882H^-associated diseases.

## Significance

DNMT3A R882H (DNMT3A^R882H^) mutation is a hot-spot mutation in acute myeloid leukemia and developmental disorders. Previous studies have identified that DNMT3A^R882H^ promotes the formation of high-order protein oligomers, giving rise to a dominant-negative effect on DNMT3A-mediated DNA methylation. This study reports the structure and mechanism for the dynamic assembly of wild-type DNMT3A homo-oligomer and the filamentous DNMT3A^R882H^ polymer, revealing a role of the autoinhibition, mediated by regulatory Pro-Trp-Trp-Pro and ATRX-DNMT3-DNMT3L domains, in potentiating the filamentous assembly of DNMT3A^R882H^. Disrupting the autoinhibitory interaction facilitates the transition of DNMT3A^R882H^ toward low-order oligomeric assembly. This study opens a window for development of effective strategies against DNMT3A^R882H^-associated diseases.

DNA methylation in mammals plays an important role in regulating gene expression, genome stability, and cell fate determination (1–4). Establishment of mammalian DNA methylation is mainly mediated by de novo DNA methyltransferases DNMT3A and DNMT3B (5), both of which are multidomain proteins, containing a C-terminal methyltransferase (MTase) domain preceded by a Pro-Trp-Trp-Pro (PWWP) domain that recognizes di- or trimethylated histone H3 lysine 36 (H3K36me2/3) (6–9) and an ATRX-DNMT3-DNMT3L (ADD) domain that reads the unmodified histone H3 lysine 4 (H3K4me0) (10, 11). In addition, the N terminus of the isoform 1 of DNMT3A (DNMT3A1) recognizes nucleosomes with monoubiquitylation of histone H2A lysine 119 (H2AK119ub1) in differentiated cells (9, 12), whereas DNMT3A isoform 2 (DNMT3A2), lacking the N-terminal 223 amino acids, is predominantly associated with euchromatin in germ cells, embryonic stem cells, and human cancers (12–14). Mutation of DNMT3A has been associated with acute myeloid leukemia (AML) (15, 16), paraganglioma (17), myelodysplastic syndromes (MDS) (15, 18), Tatton-Brown-Rahman syndrome (TBRS) (19, 20), and microcephalic dwarfism (21), while mutation of DNMT3B occurs in about half of the patients with immunodeficiency, centromeric instability, facial anomalies (ICF) syndrome (5, 22–24). Therefore, structure–functional understanding of DNMT3A and DNMT3B is of interest in therapeutic intervention against diseases and developmental disorders.

It is well known that DNMT3A- and DNMT3B-mediated de novo DNA methylation is regulated by enzymatically inactive chromatin factors DNMT3-like (DNMT3L) (25–27) and DNMT3B isoform 3 (DNMT3B3) (28–30), which function to strengthen the chromatin targeting (31) and the DNA methylation activity of DNMT3A and DNMT3B (25–27, 30), and/or protect DNMT3A from protein degradation in cells (32). Previous studies have revealed that DNMT3A and DNMT3B can each form a heterotetrameric assembly with DNMT3L (33) or DNMT3B3 (14, 30), in which each DNMT3A/3B subunit heterodimerizes with DNMT3L or DNMT3B3 via a hydrophobic interface (a.k.a. FF interface) and the DNMT3A/DNMT3B subunits further homodimerize via a hydrophilic interface (a.k.a. RD interface) (14, 33–38). In addition, DNMT3A and DNMT3B form homo-oligomers in cells, with homotetramer as a dominant form, which is important for de novo DNA methylation (38–42). Our recent structural study of the DNMT3A MTase domain, as well as DNMT3B, revealed that the assembly of DNMT3A or DNMT3B homotetramer resembles that of DNMT3A–DNMT3L (36, 37, 40). Furthermore, the DNA and cofactor-binding sites of the RD interface are disordered in the outer subunits of the homotetramer, but undergo a disordered-to-ordered transition upon further oligomerization (36, 40).

DNMT3A and DNMT3B are both subject to allosteric regulation by their N-terminal domains (43–45). Previous studies from others and us have demonstrated that in the context of DNMT3A–DNMT3L complex, both the ADD and PWWP domains engage in autoinhibitory regulation of DNMT3A, linking DNMT3A activation to the PWWP–H3K36me2 and ADD–H3K4me0 interactions, respectively (46, 47). Likewise, the DNMT3B ADD domain also engages in an autoinhibitory interaction with the MTase domain in DNMT3B homo-oligomer (36). In addition, one PWWP domain of the DNMT3B homotetramer was found to interact with the outer subunit, leading to occlusion of the DNA-binding site of the latter (36). However, how the PWWP, ADD, and MTase domains of DNMT3A interplays in controlling its functional assembly and DNA methylation activity of DNMT3A has not been elucidated.

Mutation of DNMT3A R882 (in an occurrence order of R882H > R882C > R882P, R882S) represents a hot-spot mutation in AML, accounting for ~50% of all DNMT3A missense mutations (15, 16), as well as in developmental disorders such as TBRS (20) and clonal hematopoiesis (48). It has been identified as a driver mutation for leukemogenesis (13, 46, 47), leading to selective loss of methylation in CpG islands (CGIs) (39, 49, 50) and poor prognosis in older populations (15, 39). Biochemically, the DNMT3A R882H mutation was shown to impair the DNA methylation activity and flanking sequence preference of DNMT3A (37, 51–53), promote protein polymerization (37, 39, 54), and consequently, attenuate the DNA methylation activity of wild-type (WT) DNMT3A in a dominant-negative fashion (39, 55, 56). Our recent study further revealed that the DNMT3A R882H mutation strengthens the intermolecular interaction at the RD interface, leading to a shift of DNMT3A oligomeric population from tetramer to high-order polymer (37). Introducing a DNMT3B-like, oligomerization-attenuation mutation at the RD interface, i.e., R676K or R676K/M674T, alleviated the polymerization of R882H-mutated DNMT3A in vitro and partly rescued the DNA methylation defect in cells, leading to reduced growth rate of the TF1 cells (37). However, the lack of structural knowledge on homo-oligomeric DNMT3A in the context of full-length protein has limited an effort in development of therapeutic strategies against the aberrant polymerization caused by the R882H mutation.

To determine how the regulatory PWWP and ADD domains cooperate with the MTase domain of DNMT3A in governing its dynamic assembly in development and disease, we solved the cryo-EM structures of homo-oligomeric DNMT3A2 harboring the PWWP, ADD, and MTase domains, both WT (DNMT3A^WT^) and R882H mutant (DNMT3A^R882H^). The structure of DNMT3A^WT^ reveals a hexameric assembly in which four well-defined subunits are joined by two other subunits with much reduced density at one end. In contrast, DNMT3A^R882H^ forms two alternative states of filamentous assembly: in one state (state I), over 10 DNMT3A^R882H^ stack against each other via alternating FF and RD interfaces, resulting in a left-handed helical filament; in the other state (state II), two polymer chains of DNMT3A^R882H^, each containing 8 or 10 subunits, further associate into a double-stranded filament. Structural analysis of the DNMT3A^WT^ and DNMT3A^R882H^ assembly reveals that multimerization of DNMT3A is not only attributed to the well-characterized RD and FF interfaces but also by the regulatory PWWP and ADD domains, whose autoinhibitory interaction helps stabilize the intermolecular contacts. Disrupting the PWWP domain-mediated autoinhibition not only enhances the DNA methylation activity of DNMT3A^WT^ and DNMT3A^R882H^ but also promotes the aggregation-attenuation effect of DNMT3A^R882H^ by the previously characterized rescue mutation R676K. Together, this study provides a comprehensive view of the multimeric assembly of DNMT3A and the dominant-negative DNMT3A^R882H^ mutant, with important implication in development of therapeutic strategies against DNMT3A^R882H^-mediated polymerization.

## Results

### Structure of DNMT3A Homo-Oligomer.

**Fig. 1.**
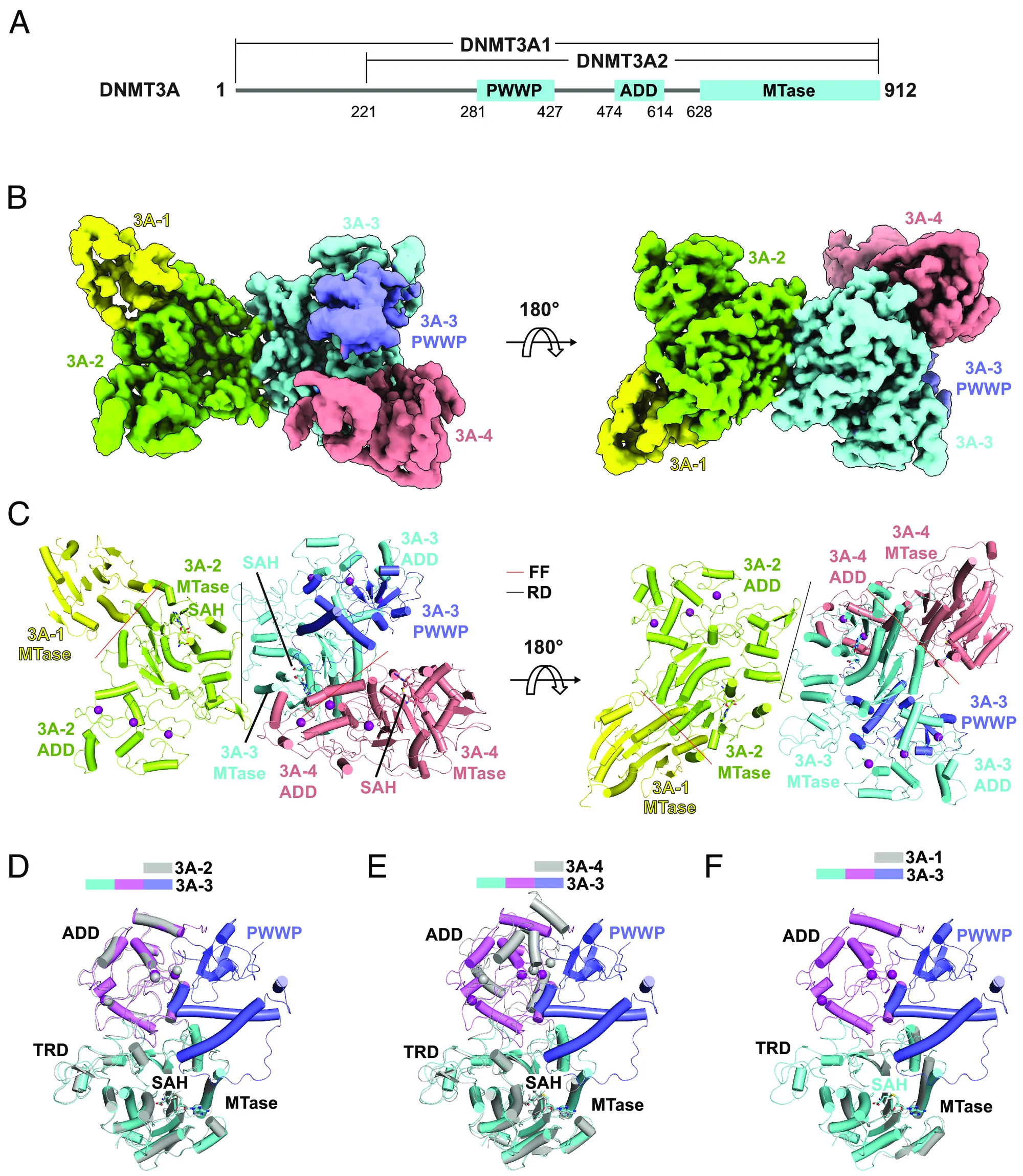
Structural overview of DNMT3A^WT^ homo-oligomer. (*A*) Domain architecture of DNMT3A1 and DNMT3A2. (*B*) Two opposite views of the DeepEMhancer-sharpened density map for DNMT3A^WT^ homotetramer, with individual subunits color coded. The PWWP domain of the 3A-3 subunit is colored slate. (*C*) Two opposite views of the atomic structure of DNMT3A^WT^ homotetramer. The FF and RD interfaces are indicated by red and black lines. (*D*–*F*) Structural overlay of the 3A-3 subunit with the 3A-2 subunit (*D*), the 3A-4 subunit (*E*), and the 3A-1 subunit (*F*). Note that the S-adenosyl-homocysteine (SAH) molecule is lacking in the target recognition domain (TRD)-disordered 3A-1 subunit.

To investigate how homo-oligomeric DNMT3A is assembled, we focused on a fragment of DNMT3A2 (residues 281 to 912) for structure determination (Fig. 1*A*). The cryo-EM structure of DNMT3A^WT^ was solved at 3.66-Å overall resolution, revealing a hexameric assembly (*SI Appendix*, Fig. S1 and Table S1). The hexamer is dominated by four subunits with relatively strong density (denoted as 3A-1, 3A-2, 3A-3, and 3A-4 herein), joined by two subunits (3A-5 and 3A-6) with much reduced density at one end (*SI Appendix*, Fig. S2 *A* and *B*). To improve map quality, we further performed local refinement for the 3A-1/3A-2/3A-3/3A-4 tetramer, resulting in a density of 3.58-Å resolution (Fig. 1 *B* and *C* and *SI Appendix*, Fig. S1*H*). The density map for the tetramer permits modeling of the PWWP (resolution of 6 to 7 Å), ADD (resolution of 4.0 to 5.5 Å), and MTase (resolution of 3 to 4 Å) domains of the 3A-3 subunit, the ADD (resolution of 4 to 6 Å) and MTase (resolution of 3 to 4 Å) domains for the 3A-2 and 3A-4 subunits, and the MTase domain (resolution of 5 to 6 Å) of the 3A-1 subunit (*SI Appendix*, Fig. S2 *C*–*K*). For analysis of the domain interaction of DNMT3A, we focused on the 3A-3 subunit for structural characterization of the intramolecular interaction of DNMT3A^WT^ in this study.

Structural comparison of the DNMT3A^WT^ tetramer with the previously reported tetrameric DNMT3A MTase (DNMT3A^MTase^, PDB 8TDR) (37) reveals a similar fashion of oligomeric assembly, mediated by alternating FF and RD interfaces (*SI Appendix*, Fig. S3 *A*–*E*). In line with the previous notion that folding of the substrate binding sites of DNMT3A/3B, including the TRD and S-adenosyl-methionine (SAM)-binding pocket, is coupled with formation of the RD interface (36, 37), the TRD and SAM pocket are well folded in those DNMT3A subunits with intact RD interface in the context of hexamer: 3A-2, 3A-3, and 3A-4 (*SI Appendix*, Fig. S2*A*); as a result, all three subunits harbor a SAH molecule, byproduct of SAM (Fig. 1 *C*–*E* and *SI Appendix*, Fig. S2*D*). In contrast, the TRD in the 3A-1 subunit becomes disordered due to the disruption of the RD interface (Fig. 1 *C* and *F* and *SI Appendix*, Fig. S3*E*).

### The Structure of DNMT3A^WT^ Homo-Oligomer Reveals an Autoinhibitory State.

**Fig. 2.**
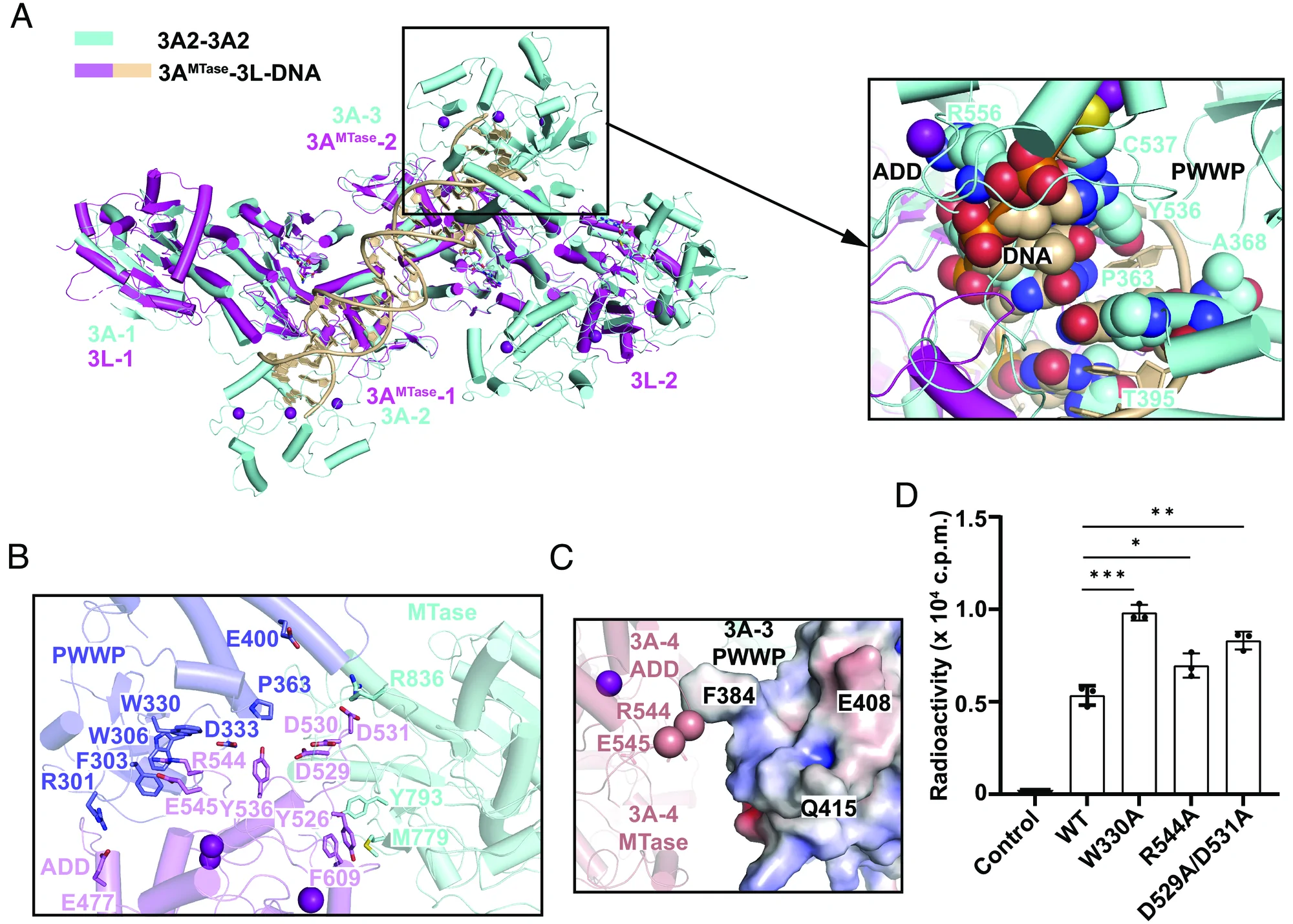
Structural and biochemical analysis of DNMT3A^WT^ homo-oligomer. (*A*) Structural overlay between the DNMT3A^WT^ homotetramer and the complex of DNMT3A^MTase^–DNMT3L–DNA (PDB 5XY2), with steric clash between the residues of DNMT3A^WT^ and the DNA shown in sphere representation in the expanded view. (*B*) Close-up view of the trilateral interaction between the PWWP, ADD, and MTase domains of the 3A-3 subunit. (*C*) Close-up view of the spatial proximity between the 3A-3 PWWP domain in electrostatic surface representation and the ADD domain of the 3A-4 subunit in ribbon representation, with select contact sites labeled. The contact sites on the 3A-4 ADD domain are shown as sphere representation of the Cα atoms and the 3A-3 PWWP domain are shown in electrostatic surface representation. (*D*) In vitro DNA methylation analysis of WT and mutant DNMT3A2 on a CpG DNA. The statistical analysis used two-tailed Student’s *t* test. Data are mean ± SD (n = 3 biological repeats). *P < 0.05; **P < 0.01; ***P < 0.001.

Structural alignment of the DNMT3A homo-oligomer with the previously reported DNMT3A^MTase^–DNMT3L bound to CpG DNA (PDB 5YX2) (57) reveals high structural similarity, with rmsd of 1.12 Å over 542 aligned Cα atoms (Fig. 2*A*). However, it is apparent that the DNA molecule in the DNMT3A^MTase^–DNMT3L–DNA complex is in steric clash with residues from the ADD domains of 3A-2 and 3A-3 (e.g., Y536, C537, and R556) and the PWWP domain of 3A-3 (e.g., P363, A368, and T395) in DNMT3A^WT^ homo-oligomer, suggesting that the DNA-free DNMT3A^WT^ homo-oligomer adopts an autoinhibitory conformation (Fig. 2*A*). Detailed structural analysis reveals that such an autoinhibitory state is mediated by the trilateral intramolecular interaction between the PWWP, ADD, and MTase domains: For instance, within the 3A-3 subunit, the ADD residues D529, D530, and D531 engage in electrostatic contacts with residue R836 from the CpG-recognition loop of the TRD (57). The ADD–MTase interaction is further supported by the van der Waals contacts involving ADD residues Y526, Y536, and F609 and MTase residues M779 and Y793 (Fig. 2*B*). Note that the ADD domain is similarly positioned in the 3A-2 subunit but undergoes slight rotation in the 3A-4 subunit (Fig. 1 *D* and *E*), suggesting that the ADD domain-mediated autoinhibition may be influenced by the integrity of the RD interface. Furthermore, the PWWP domain of the 3A-3 subunit interacts with its ADD domain, with the H3K36me2-binding pocket (residues F303, W306, W330, and D333) in the PWWP domain (58) harboring the sidechain guanidinium group of ADD residue R544 (Fig. 2*B*). Other PWWP–ADD interactions of the 3A-3 subunit are mediated by electrostatic or van der Waals contacts involving PWWP residues R301, F303, and P363 and ADD residues E477, Y536, and E545. In addition, PWWP residue E400 and TRD residue R836 are positioned in proximity for electrostatic contact (Fig. 2*B*). Together, these intramolecular interactions led to occlusion of the R836-residing TRD loop from DNA binding, resulting in an autoinhibitory state.

Detailed analysis of the DNMT3A homo-oligomer further reveals that the autoinhibitory interaction between the PWWP, ADD, and MTase domains of the 3A-3 subunit places the PWWP domain in a position spatially proximate to the ADD domain of the 3A-4 subunit, with a modest contact interface (buried surface area of ~131 Å^2^) involving residues of 3A-3 F384, E408, and Q415 and 3A-4 R544 and E545 (Fig. 2*C*). Conceivably, such intermolecular contact between the 3A-3 and 3A-4 subunits may further contribute to the oligomeric and autoinhibitory assembly of DNMT3A^WT^. Consistent with the structural observations, our in vitro DNA methylation analysis reveals that introducing the W330A mutation at the PWWP–ADD interface, the R544A mutation at the PWWP–ADD and 3A-3/3A-4 interfaces, and the D529A/D531A double mutation at the ADD–MTase interface all led to a notable increase in the DNA methylation activity of DNMT3A^WT^ on a CpG DNA (Fig. 2*D*).

It is worth noting that the autoinhibitory state of the DNMT3A^WT^ homo-oligomer resembles the previously reported autoinhibitory state of DNMT3A2–DNMT3L heterotetramer (PDB 9PRW) (47). Structural overlay of DNMT3A^WT^ tetramer with the DNMT3A2–DNMT3L complex reveals high similarity, giving RMSD of 1.0 Å over 997 aligned Cα atoms (*SI Appendix*, Fig. S3*F*). Of note, the DNMT3A2 homotetramer and DNMT3A2–DNMT3L heterotetramer resort to the same set of residues for the trilateral interaction between the PWWP, ADD, and MTase domains (*SI Appendix*, Fig. S3*F*), suggesting such an autoinhibitory interaction represents an intrinsic mechanism of DNMT3A regulation in the context of both homo-oligomeric and hetero-oligomeric assemblies.

### Structure of DNMT3A2^R882H^ Polymer.

**Fig. 3.**
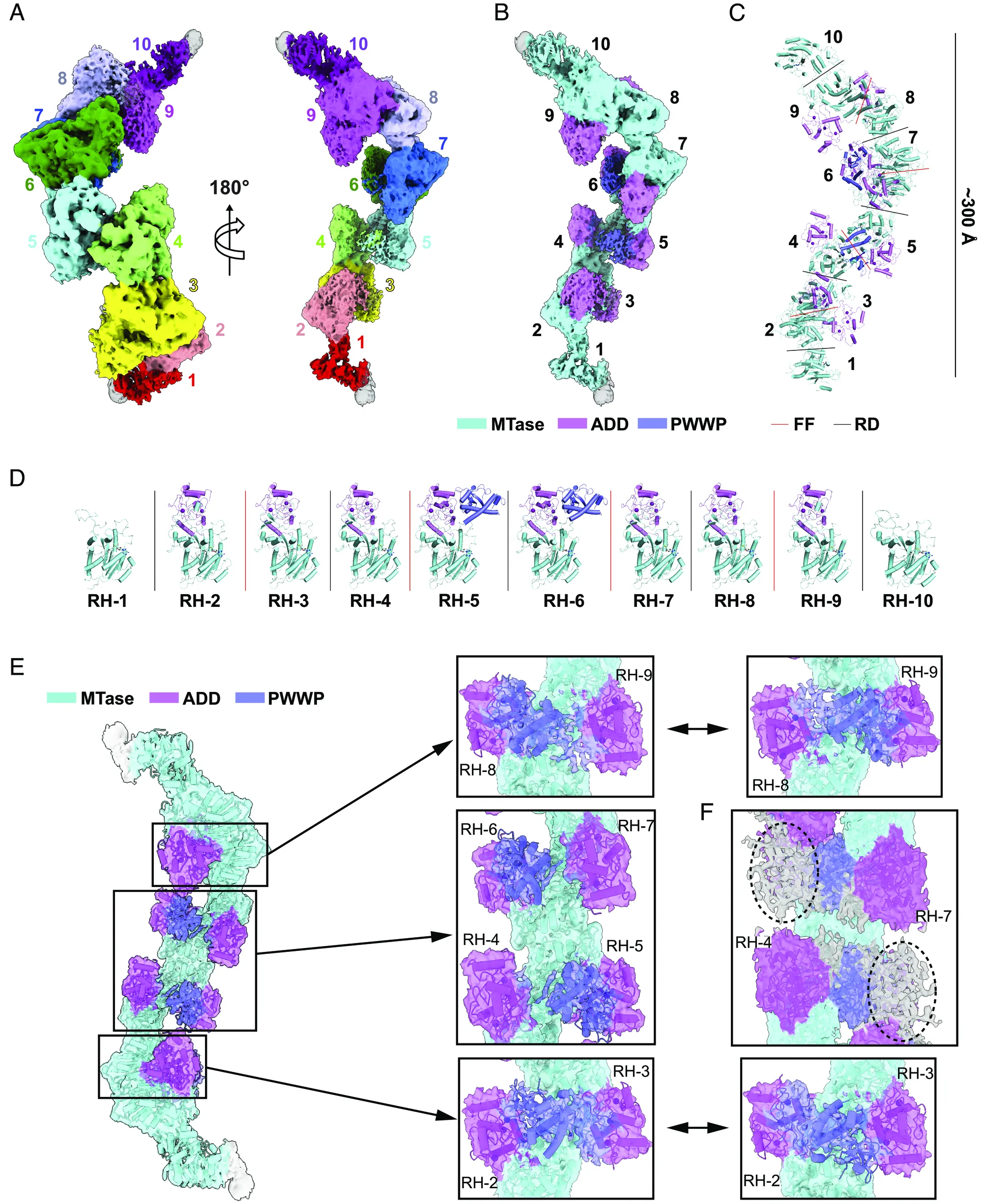
Cryo-EM structure of state-I DNMT3A^R882H^ polymer. (*A*) Two opposite views of the density map of state-I DNMT3A^R882H^ polymer at contour level 0.17, with individual subunits color coded and numbered sequentially. (*B* and *C*) Density map at contour level 0.17 (*B*) and atomic structure (*C*) of state-I DNMT3A^R882H^ polymer, the MTase, ADD, and PWWP domains colored in aquamarine, violet, and slate, respectively. The length of the polymer is indicated. (*D*) Atomic structures of individual subunits of state-I DNMT3A^R882H^ polymer. (*E*) Density map of the state-I DNMT3A^R882H^ polymer at contour level of 0.17, overlaid with the atomic model, with the segments of RH-8 and RH-9 (*Top*), RH-4, RH-5, RH-6, and RH-7 (*Middle*), and RH-2 and RH-3 (*Bottom*) shown in the expanded views. For the RH-8/RH-9 or RH-2/RH-3 pair, the density for the PWWP domain can be assigned to either subunit, likely reflecting a population average between the two alternative states. (*F*) Close-up view of the density map for the RH-4 and RH-7 subunits at contour level of 0.11. The unmodeled density corresponding to the PWWP domains are colored gray.

**Fig. 4.**
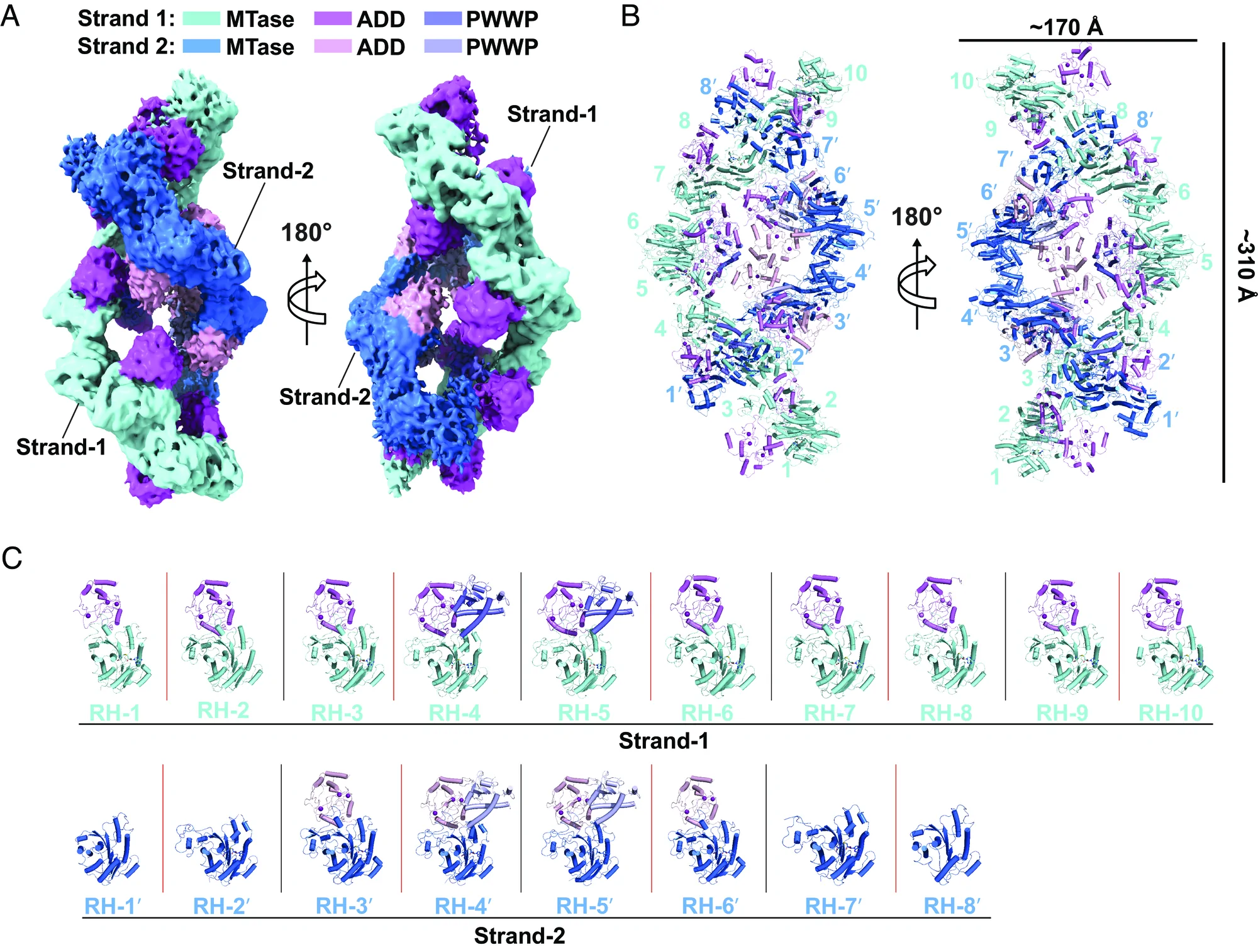
Cryo-EM structure of state-II DNMT3A^R882H^ polymer. (*A* and *B*) Density map at contour level of 0.13 (*A*) and atomic structure (*B*) of state-II DNMT3A^R882H^ polymer, with the MTase, ADD, and PWWP domains of strand-1 colored in aquamarine, violet, and slate, respectively, and the MTase, ADD, and PWWP domains of strand-2 are colored in marine, light pink, and light blue, respectively. The length and width of the polymer are indicated. (*C*) Atomic structures of individual subunits of state-II DNMT3A^R882H^ polymer.

To gain a comprehensive view on how the DNMT3A R882H mutation affects its assembly, we further performed single-particle cryo-EM characterization of DNMT3A^R882H^. After 3D classification, two classes of the DNMT3A^R882H^ particles with well-defined structural features were identified: In one class (state I), DNMT3A^R882H^ polymerizes into a left-handed helical filament, in which more than 10 DNMT3A^R882H^ subunits stack against each other via the alternating RD and FF interfaces (Fig. 3); in the other state (state II), two left-handed DNMT3A^R882H^ polymers are twisted face-to-face to form a double-stranded filament, resulting in a left-handed DNA duplex-like shape: The MTase domains of DNMT3A^R882H^ stack into the backbone, positioning the regulatory ADD and PWWP domains inside the evenly spaced groove (Fig. 4). The density of state-I and state-II DNMT3A^R882H^ polymers was refined to 4.19-Å and 5.04-Å overall resolution, respectively (*SI Appendix*, Fig. S4 and Table S1).

Analysis of the density for state-I filament reveals substantial variation of local resolution: The density of the central subunits shows the highest resolution, which then gradually decreases as the polymer chain extends toward the two ends (*SI Appendix*, Fig. S4*D*). This observation suggests an entropy–enthalpy interplay in governing the assembly of DNMT3A^R882H^. For structural characterization, we focused on 10 of the DNMT3A^R882H^ subunits (RH-1 to RH-10) with resolvable density for structural analysis but left the continuing subunits with much reduced density at the two ends unmodeled (Fig. 3 *A*–*D*). The 10 DNMT3A^R882H^ subunits assemble into a left-handed helix with a length of ~300 Å. We were able to model the ADD and MTase domains for the RH-2 to RH-8 subunits of DNMT3A^R882H^ subunits, but the MTase domain only for RH-1 and RH-10 (Fig. 3*D*). Additionally, the PWWP domains of RH-5 and RH-6 were defined, positioned between the ADD domains of the RH-4/RH-5 pair and the ADD domains of the RH-6/RH-7 pair, respectively (Fig. 3 *D* and *E*). Note that the spacing between the ADD domains of the RH-4/RH-5 or RH-6/RH-7 pair may only accommodate one PWWP domain at one time, suggesting that the unmodeled PWWP domains of the RH-4 and RH-7 subunits are likely dynamically disordered. Consistent with this notion, inspection of state-I DNMT3A^R882H^ density at a lower contour level reveals residual density proximate to the ADD domain of RH-4 or RH-7, which likely corresponds to their respective PWWP domains (Fig. 3*F*). The density for the PWWP domain was also observed for the RH-2/RH-3 and RH-8/RH-9 pairs. However, due to the ambiguity of the density, we were unable to assign the subunit with which the density was associated, raising the possibility that such density may represent a population average of two alternative conformational states (Fig. 3*E*).

The density for state-II DNMT3A^R882H^ permitted us to model 10 DNMT3A^R882H^ subunits (RH-1 to RH-10) for one strand (strand 1) and 8 subunits (RH-1′ to RH-8′) for the other (strand 2) (Fig. 4 *A*–*C*). Notably, the two chains of state-II DNMT3A^R882H^ are associated with differential local resolution, with the resolution ranging from 3 to 7 Å for strand 1 and from 7 to 12 Å for strand 2 (*SI Appendix*, Fig. S4*G*), suggesting substantial conformational dynamics in strand association. Similar to what was observed for state-I DNMT3A^R882H^ polymer, the highest local resolution was observed for the central subunits of the two strands (e.g., RH-4/RH-5/RH-6 in strand 1 and RH-4′/RH-5′ in strand 2); it then gradually decreases as the chains extend toward the two ends of each strand (*SI Appendix*, Fig. S4*G*). The ADD and MTase domains were modeled for all subunits of strand 1 as well as the RH-3′ to RH-6′ subunits of strand 2 (Fig. 4*C* and *SI Appendix*, Fig. S5*A*). In addition, the PWWP domains were modeled for the two RD interface-linked central subunits of each chain (RH-4 and RH-5 for strand 1 and RH-4′ and RH-5′ for strand 2) (Fig. 4*C*). On the other hand, we were only able to model the MTase domain for the RH-1′, RH-2′, RH-7′ and RH-8′ subunits of strand 2 (Fig. 4*C*), presumably due to the high conformational dynamics of these subunits.

Structural alignment of state-I DNMT3A^R882H^ with strands 1 and 2 of state-II DNMT3A^R882H^ reveals high similarity, giving RMSD of 0.87 Å and 0.76 Å over 3,459 and 2,184 aligned Cα atoms, respectively (*SI Appendix*, Fig. S5 *B* and *C*). Accordingly, the helical assembly of state-II polymer extends in a similar geometry to that of state-I polymer, with a length of ~310 Å and width of ~170 Å (Fig. 4*B*).

### Multipronged Domain Interactions Underpin DNMT3A^R882H^ Polymerization.

**Fig. 5.**
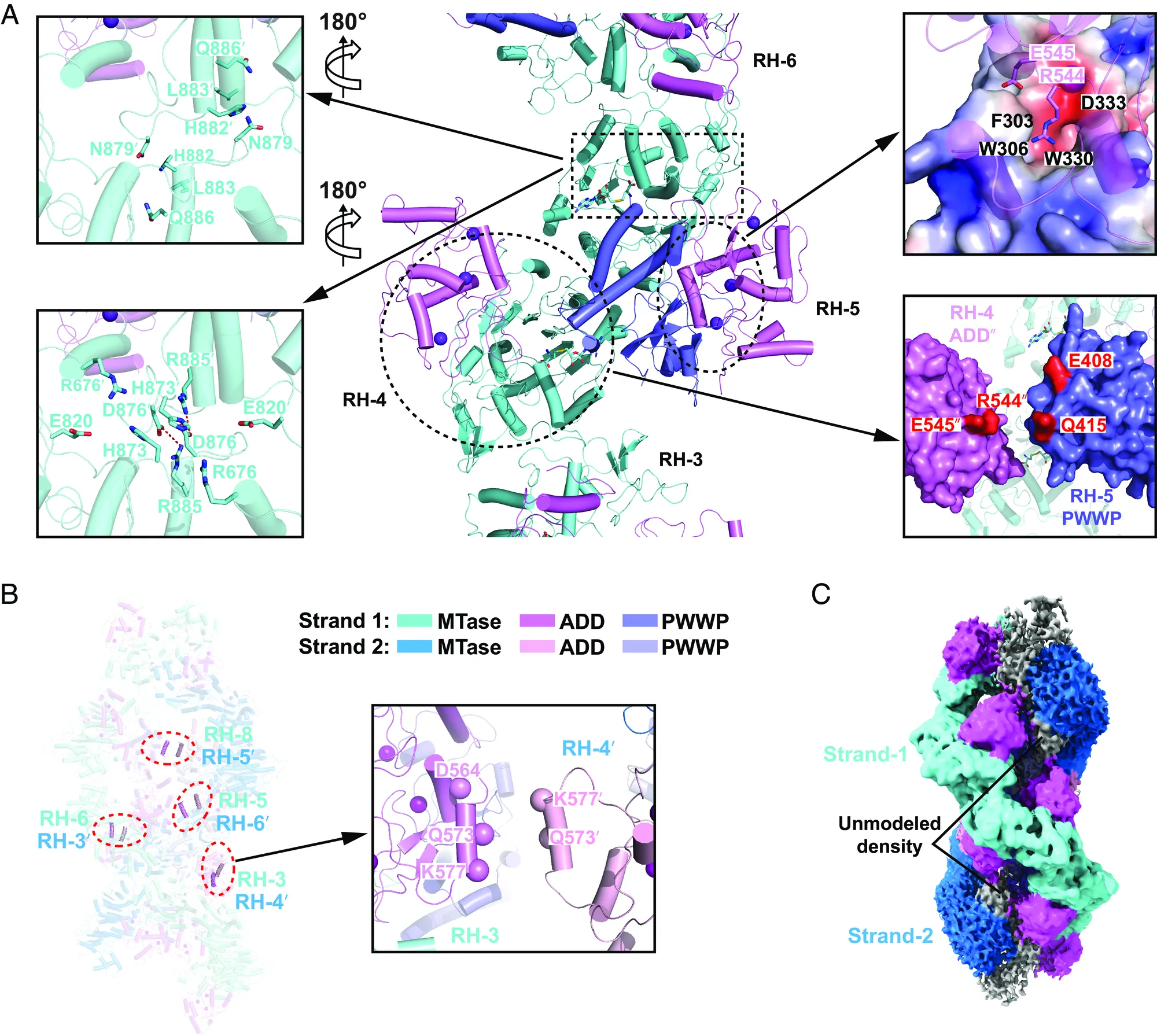
Structural analysis of state-I and state-II DNMT3A^R882H^ polymers. (*A*) Close-up view of the interaction of the RH-5 subunit with the RH-4 and RH-6 subunits. The RD interface between the RH-5 and RH-6 are shown in the expanded views on the left, with the H882-centered region on *Top* and the rest at the *Bottom*. The intramolecular PWWP–ADD interaction is shown on the top right, with ADD residues R544 and E545 shown in stick representation and the interacting residues of the PWWP domain shown in electrostatic surface representation. The intermolecular contact between the RH-5 PWWP domain (slate) and the RH-4 ADD domain (violet) is shown on the bottom right, with interacting residues colored red. (*B*) Cartoon representation of state-II DNMT3A^R882H^ polymer, with the helical packing interactions between the two strands dot circled and/or highlighted in the expanded view (for the RH-3/RH-4′ pair). (*C*) Density map of the state-II DNMT3A^R882H^ polymer at contour level of 0.09, highlighting the density blob (gray) corresponding to the unmodeled PWWP domains.

Next, we asked how the assembly of DNMT3A^R882H^ is related to that of DNMT3A^WT^. Structural alignment of state-I DNMT3A^R882H^ polymer with DNMT3A^WT^ homotetramer reveals high similarity, with rmsd of 0.50 Å over 776 aligned Cα atoms for the RH-2/RH-3/RH-4/RH-5 segment, 0.68 Å over 810 aligned Cα atoms for the RH-4/RH-5/RH-6/RH-7 segment, and 0.52 Å over 781 aligned Cα atoms for the RH-6/RH-7/RH-8/RH-9 segment (*SI Appendix*, Fig. S6 *A*–*C*). Notably, the PWWP and ADD domains of DNMT3A^R882H^ engage in autoinhibitory interaction in a similar fashion to that for DNMT3A^WT^, with the H3K36me2-binding pocket in the PWWP domain anchoring the side chain of ADD residue R544 within the same subunit (Fig. 5 *A*, *Top Right*). As with that in DNMT3A^WT^, the RD interface of DNMT3A^R882H^ is mainly mediated by the salt bridges of the reciprocal R885/D876′ (residues from the RD interface-related subunit is denoted by prime symbol) pairs, reinforced by additional electrostatic and/or van der Waals contacts involving residues R676, E820, and H873 (Fig. 5*A* and *SI Appendix*, Fig. S6*A*). These observations highlight strong structural similarity between DNMT3A^R882H^ and DNMT3A^WT^. Nevertheless, the replacement of DNMT3A^WT^ R882 with a histidine in DNMT3A^R882H^ leads to increased sidechain packing between site 882 and residue N879′ from the RD interface-related subunit, as well as van der Waals contact with residues L883 and Q886 within the same subunit, as previously observed for the tetrameric DNMT3A^MTase^ (Fig. 5 *A*, *Left*) (37). Consequently, the RD interface of DNMT3A^R882H^ involves more extensive intermolecular contacts than that of DNMT3A^WT^ (buried surface area of ~833 Å^2^ in DNMT3A^R882H^ vs. ~715 Å^2^ in DNMT3A^WT^). It is worth noting that, in addition to the RD and FF interfaces, the multimeric assembly of DNMT3A^R882H^ is further supported by modest domain contact between the PWWP domain of one subunit (e.g., RH-5) and the ADD domain of the FF interface-related neighboring subunit (e.g., RH-4) (Fig. 5*A*), as observed for the DNMT3A^WT^ homo-oligomer (Fig. 2*C*).

Structural analysis of state-II DNMT3A^R882H^ filament reveals that the association between the two strands of DNMT3A^R882H^ polymer is underpinned by four pairs of antiparallel helical packing interactions, involving a short helix (residues G570-K577) from the ADD domains of RH-3/RH-4′, RH5/RH-6′, RH-6/RH-3′ and RH-8/RH-5′, which together give rise to buried surface area of ~350 Å^2^ (Fig. 5*B*). In addition, analysis of the density map of state-II filament at a lower contour reveals a blob of unmodeled density clustered at the core region of the filament, likely arising from those unmodeled PWWP domains, which may provide transient intermolecular contact between the two chains (Fig. 5*C*).

### The PWWP and ADD-Mediated Domain Interaction Facilitates DNMT3A^R882H^ Polymerization.

**Fig. 6.**
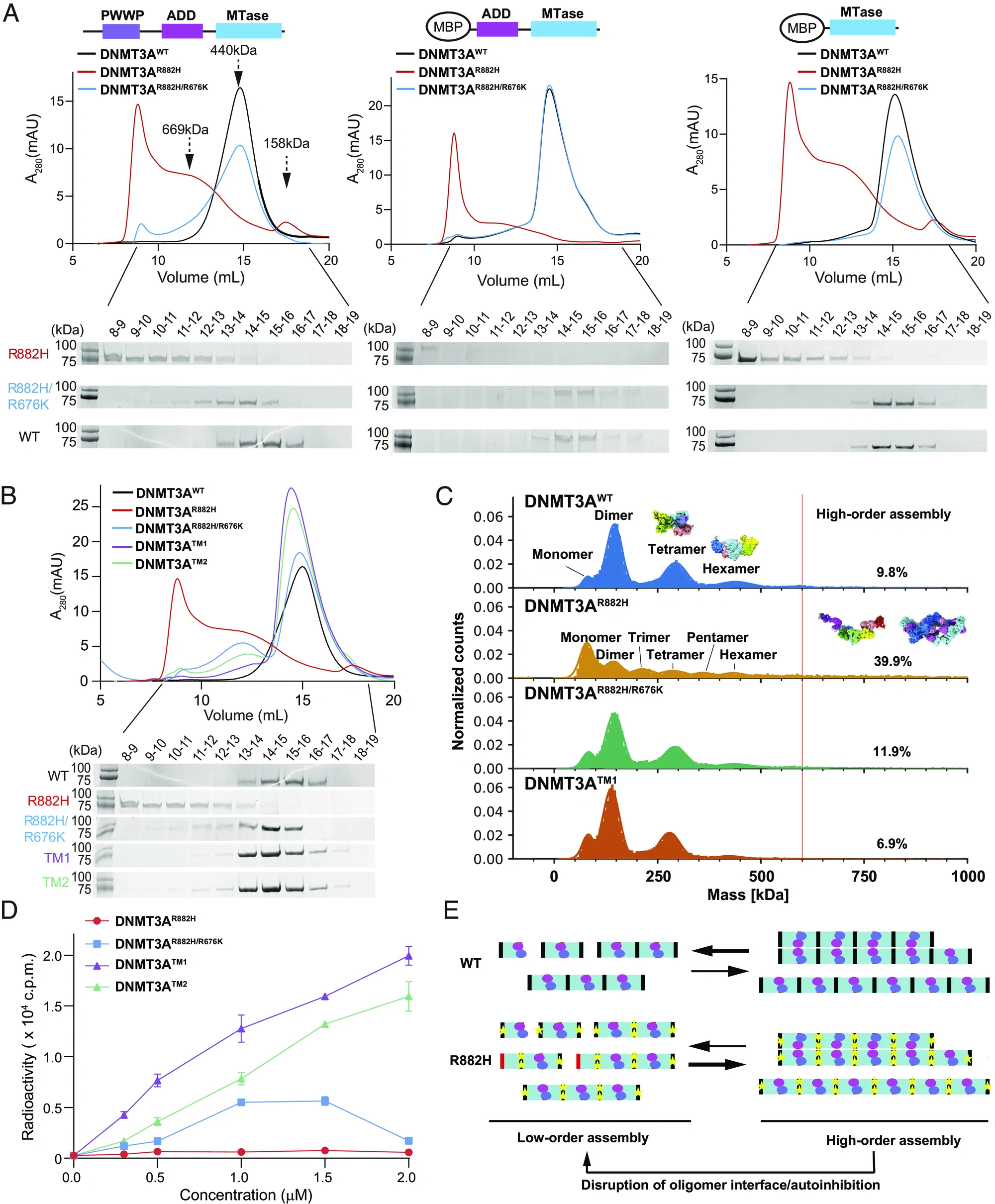
Biochemical analysis of DNMT3A^R882H^ polymerization. (*A*) Size-exclusion chromatography analysis of the PWWP–ADD–MTase (*Left*), MBP-tagged ADD–MTase (*Middle*), and MBP-tagged MTase (*Right*) fragments of WT and mutant DNMT3A. The SDS-PAGE images of the fractions with various elution volumes are shown beneath. The elution volumes for molecular weight standards (Thyroglobulin: 669 kDa, Ferritin: 440 kDa, Adolase: 158 kDa) are indicated by arrows. (*B*) Size-exclusion chromatography analysis of the PWWP–ADD–MTase fragment of DNMT3A^WT^, DNMT3A^R882H^, DNMT3A^R882H/R676K^, DNMT3A^R882H/R676K/W330A^ (TM1) and DNMT3A^R882H/R676K/R544A^ (TM2). (*C*) Mass photometry analysis of the DNMT3A PWWP–ADD–MTase fragment, WT or mutant. The peaks corresponding to the monomers, dimers, tetramers, and hexamers of DNMT3A^WT^ are indicated. The peak area corresponding to molecular weight of >600 kDa was analyzed to assess the extent of high-order assembly. The calculated molecular weights for monomeric, dimeric, tetrameric, and hexameric DNMT3A^WT^ are 72.1 kDa, 144.3 kDa, 288.5 kDa, and 432.8 kDa, respectively. (*D*) Protein concentration-dependent DNA methylation activity of the PWWP–ADD–MTase fragment of DNMT3A^R882H^, DNMT3A^R882H/R676K^, DNMT3A^R882H/R676K/W330A^ (TM1) and DNMT3A^R882H/R676K/R544A^ (TM2) Data are mean ± SD (n = 3 biological replicates). (*E*) A model for the dynamic assembly of DNMT3A^WT^ and DNMT3A^R882H^, highlighting their distinct assembly behavior and the role of the PWWP–ADD domain in potentiating polymerization. The RD and FF interface are indicated by black and red bars, respectively. The MTase, ADD, and PWWP domains are colored in aquamarine, violet, and slate, respectively. For simplicity, only the PWWP–ADD cassettes engaging intermolecular contacts are indicated. The R882H mutation is indicated by yellow asterisk. The equilibrium between the low-order assembly and high-order assembly may be modulated via disruption of the oligomer interface and/or the autoinhibitory interaction.

The structural observations that the regulatory PWWP and ADD domain contribute to intermolecular interaction of DNMT3A^WT^ and DNMT3A^R882H^ prompt us to ask whether these domains affect the dynamic assembly of the corresponding proteins. To address this, we performed size-exclusion chromatography for various fragments of WT and mutant DNMT3A, with a loading monomeric concentration of ~8 µM. Consistent with previous observations (37, 39), DNMT3A^WT^ eluted with a volume corresponding to a molecular weight of tetramer to hexamer, whereas DNMT3A^R882H^ eluted with a volume corresponding to much higher molecular weight (>669 kDa) (Fig. 6 *A*, *Left*). Introducing the previously reported rescue mutation at the RD interface, R676K, to DNMT3A^R882H^ shifted the elution volume greatly toward that of DNMT3A^WT^, but minor population remains eluted in the high-order assembly state (Fig. 6*A*), as previously observed (37). The polymerization-attenuation effect of the R676K appears even stronger in the shorter ADD–MTase and MTase fragments: Whereas the DNMT3A^R882H^ mutation similarly led to a high-order polymeric state of these fragments, shift of the elution volume toward that of DNMT3A^WT^ by the R676K mutation appears more complete (Fig. 6 *A*, *Middle* and *Right*). Next, we introduced the mutation that disrupts the PWWP–ADD interaction, W330A or R544A, to DNMT3A^R882H^. Whereas the R544A mutation failed to affect the elution of DNMT3A^R882H^ appreciably, introducing the W330A mutation led to a slight but notable shift of the elution toward the low-order polymeric states (*SI Appendix*, Fig. S7*A*), and both mutations were found to modestly strengthen the R676K-mediated attenuation of DNMT3A^R882H^ polymerization (Fig. 6*B*).These data support the role of the PWWP–ADD interaction in stabilizing the DNMT3A^R882H^ polymers. In addition, we introduced the Q573G/A574G/A575G mutation to DNMT3A^R882H^ (resulting Q573G/A574G/A575G/R882H quadruple mutation denoted as DNMT3A^QM^ herein) to disrupt the ADD helix involved in state-II filament formation (Fig. 5*B*). Compared with DNMT3A^R882H^, DNMT3A^QM^ showed a relatively lower level of protein aggregation (*SI Appendix*, Fig. S7*B*), supporting the role of the ADD helix in the high-order filamentous assembly of DNMT3A^R882H^. Together, these observations support the structural observation that the regulatory PWWP and ADD domains contribute to the high-order polymerization of DNMT3A^R882H^.

Previous characterization of DNMT3A oligomers has indicated that they undergo a sequential oligomerization process: DNMT3A monomers first dimerize through the hydrophobic FF interface, then undergo further oligomerization through the hydrophilic RD interface, accompanied by the disorder-to-order transition of the DNA-binding site of the TRD (36–38). Consistently, our mass photometry analysis of DNMT3A^WT^ at 10 nM concentration reveals that DNMT3A^WT^ exists in a mixed population of monomers, dimers, tetramers, and hexamers, with the high molecular weight polymers (molecular weight greater than 600 kDa) accounting for 9.8% of the population (Fig. 6*C* and *SI Appendix*, Fig. S7*C*). In contrast, DNMT3A^R882H^ shows reduced population of tetramers and dimers but increased population of high molecular weight polymers (39.9%) (Fig. 6*C*). Introducing the R676K mutation to DNMT3A^R882H^ enhanced the population of tetramer and hexamer, while reducing the high molecular weight polymeric state to 11.9% (Fig. 6*C*). On the other hand, introducing the PWWP mutation W330A to DNMT3A^R882H/R676K^ led to slightly decreased population of high molecular weight polymers (6.9%) without affecting the distribution of the low-order oligomers (monomers to hexamers) appreciably, likely because the latter are already thermodynamically favorable under the low protein concentration condition (Fig. 6*C*). Together, these observations support that the PWWP and ADD domains fine-tune DNMT3A^R882H^ polymerization. In addition, our mass photometry analysis of DNMT3A^R882H^ reveals enhanced population of monomers, trimers, and pentamers, suggesting a shift of the energetic landscape between the RD and FF interfaces (Fig. 6*C*).

Finally, we performed in vitro DNA methylation assays at various concentrations of DNMT3A^WT^ and DNMT3A^R882H^. The DNA methylation activity of the PWWP–ADD–MTase fragment of DNMT3A^WT^ first increased as the protein concentration increased from 0 to 1.0 µM, then decreased as the protein concentration increased from 1.0 to 2.0 µM (*SI Appendix*, Fig. S7*D*), consistent with a previous observation (54). In contrast, the activity for the autoinhibition-disrupting mutant (W330A or R544A) or the DNMT3A MTase domain underwent monotonic increase as the protein concentration increased (*SI Appendix*, Fig. S7*D*). Likewise, whereas the activity of DNMT3A^R882H^ or DNMT3A^R882H/R676K^ failed to increase continuously after the concentration reached higher than 1.0 µM, introducing the W330A and R544A mutation to DNMT3A^R882H/R676K^ (TM1: R882H/R676K/W330A; TM2: R882H/R676K/R544A) led to monotonic activity increase with increasing protein concentration (Fig. 6*D*). These observations support the notion that disruption of the autoinhibitory interactions of the PWWP and ADD domains provides a way to modulate the DNA methylation activity of DNMT3A^R882H^. It is worth noting that disrupting state-II filament by the Q573G/A574G/A575G mutation failed to increase the DNA methylation activity of DNMT3A^R882H^ appreciably, suggesting a dominant role of state-I filament in activity inhibition of DNMT3A^R882H^ (*SI Appendix*, Fig. S7*E*).

## Discussion

The AML hot-spot DNMT3A R882H mutation, with its dominant-negative effect in DNA methylation and grave impact on development and disease, has become an attractive therapeutic target in fighting against AML and developmental disorders. However, development of small-molecule inhibitors against this mutation has been challenged by the lack of a deep understanding of its structure and function. This study provides a comprehensive view of the structural basis underlying the dynamic assembly of DNMT3A^WT^ and DNMT3A^R882H^, uncovering the intricate interplay between the regulatory and MTase domains governing the DNA methylation activity and polymerization of DNMT3A and its disease mutant.

First of all, this study provides a framework for the homo-oligomeric assembly of DNMT3A^WT^ and DNMT3A^R882H^, revealing the autoinhibitory state of each DNMT3A^WT^ or DNMT3A^R882H^ subunit as the repeating unit of the multimeric assembly and an enthalpy–entropy interplay in governing the process of multimerization (Fig. 6*E*). Importantly, we found that the autoinhibitory interaction of the regulatory PWWP and ADD domains with the MTase domain place the former in a position to facilitate the multimeric assembly of DNMT3A. For DNMT3A^R882H^, such conformational stabilization by the PWWP and ADD domains further encourages the polymerization process, powered by the R882H-enhanced intermolecular interactions at the RD interface, resulting in formation of large filaments composed of either single- or double-stranded DNMT3A^R882H^ polymers (Fig. 6*E*).

The fact that the autoinhibitory state of DNMT3A facilitates its oligomeric assembly further suggests that the assembly of DNMT3A is subject to influence by chromatin environment. Given that the autoinhibitory state of DNMT3A is coupled with occlusion of the H3K36me2-binding site of the PWWP domain and the H3K4me0-binding site of the ADD domain, it is conceivable that the PWWP–H3K36me2 and ADD–H3K4me0 readout would promote repositioning of the PWWP and ADD domains respectively, as previously observed for the DNMT3A2–DNMT3L complex (47), which would consequently affect the DNMT3A assembly. For DNMT3A^R882H^, the filamentous assembly presumably raises the energy barrier for the transition from an inactive state to an active state. How the multimeric assembly of DNMT3A or DNMT3A^R882H^ interplay with their chromatin interaction in cells await further investigation.

Increasing evidence has pointed to a complex interplay between protein polymerization and flanking sequence preference of DNMT3A^R882H^ in creating genomic hypomethylation. On one hand, the R882H mutation promotes the polymerization of DNMT3A via strengthening the intermolecular contacts at the RD interface (Fig. 5*A*), leading to reduced DNA methylation activity of DNMT3A^R882H^ (37, 39, 54). Conceivably, under the condition where DNMT3A^WT^ and DNMT3A^R882H^ are equally mixed, as what occurs in most normal karyotype AML samples with the R882H mutation (39), the stable association of a DNMT3A^R882H^ filament may permit stochastic incorporation of DNMT3A^WT^ subunits, thereby dominant-negatively suppressing the DNA methylation activity of DNMT3A^WT^. In support of this notion, our recent study showed that fine-tuning the RD interface through introducing the DNMT3B-converting mutations R676K and/or M674T, which are positioned well away from the DNA-contact surface or the PWWP–ADD autoinhibition interface, led to a shift of DNMT3A^R882H^ population toward the tetramer in vitro. In cells, introducing these aggregation-attenuating mutations partly rescued the DNA methylation at the promoter regions, and consequently, reduced the transformation potential of DNMT3A R882H and R882C mutations in TF1 cells (37). On the other hand, the observation that the DNMT3A R882H mutation led to selective hypomethylation at the targets of polycomb repressive complex 2 and the CGT motif also links the pathological consequence of DNMT3A^R882H^ to its altered flanking sequence preference (37, 50, 52, 53). Intriguingly, this study found that the autoinhibitory PWWP and ADD domains contribute to the filament formation of DNMT3A^R882H^, adding another level of regulation of DNMT3A^R882H^ assembly and activity. To clarify the role of DNMT3A^R882H^ autoinhibition in disease progression, future studies are warranted to test whether disruption of the DNMT3A^R882H^ autoinhibition, either via removal of the PWWP–ADD domains or via introducing the autoinhibition-disrupting mutation (e.g., W330A), might influence the dominant-negative behavior of the R882H mutation in cells.

Finally, this study provides insight into development of small-molecular inhibitors against DNMT3A R882H-associated diseases. Whereas the structural basis for the R882H mutation-promoted intermolecular interaction at the RD interface has been elucidated (37), development of small-molecule inhibitors targeting the RD interface is challenged by the lack of potential ligand-binding sites around the region. In this regard, this study reveals that the PWWP and ADD domains further contribute to the protein polymerization of R882H, which makes these two regulatory domains a possible target for reversing the DNMT3A R882H inhibition. Whether it is possible to develop small molecules that can perturb the DNMT3A autoinhibition, thereby influencing the high-order assembly and DNA methylation activity of DNMT3A^R882H^, awaits further investigation.

## Methods

### Protein Expression and Purification.

The DNA sequence encoding a C-terminal fragment of human DNMT3A (281 to 912) was cloned into an in-house bacterial expression vector containing an N-terminal hexahistidine–maltose-binding protein (His_6_–MBP) tag followed by a tobacco etch virus (TEV) protease cleavage site, as previously described (37, 47). The expression plasmid was transformed into *Escherichia coli* BL21(DE3) RIL cells for protein expression. The transformed cells were cultured at 37 °C until OD_600_ of ~1.0 was reached. Protein expression was induced by 0.07 mM isopropyl β-D-1-thiogalactopyranoside in the presence of 50 µM ZnCl_2_. The cells continued to grow at 16 °C overnight. After harvest by centrifugation, the cells were resuspended in lysis buffer containing 50 mM Tris–HCl (pH 8.0), 1 M NaCl, 25 mM imidazole, 10% (v/v) glycerol, 10 µg mL^−1^ DNase I, and 1 mM phenylmethylsulfonyl fluoride (PMSF), and subject to disruption and clarification. The supernatant was applied to Ni^2+^-affinity chromatography, followed by removal of the His_6_-MBP tag via TEV protease cleavage, and ion-exchange chromatography using a HiTrap Heparin HP column (Cytiva). Finally, the protein was purified through size-exclusion chromatography on a Superdex 200 pg 16/600 column (Cytiva) equilibrated in 20 mM HEPES-NaOH (pH 7.2), 250 mM NaCl, 5% (v/v) glycerol, and 5 mM DTT. Mutants of the DNMT3A fragment were constructed via site-directed mutagenesis and produced using the same approach as that for WT protein. Proteins were concentrated, flash-frozen in liquid nitrogen, and stored at −80 °C.

In addition, the DNA sequences for DNMT3A MTase (residues 628 to 912) and DNMT3A ADD–MTase (residues 455 to 912) were each cloned into the above-mentioned expression vector. The proteins were expressed and purified via Ni^2+^–affinity chromatography, ion-exchange chromatography on a HiTrap Heparin HP column, and size-exclusion chromatography on a Superdex 200 pg 16/600 column, as described above. The final protein sample was stored in 20 mM Tris–HCl (pH 7.2), 250 mM NaCl, and 5 mM DTT. The N-terminal His_6_–MBP tag was retained throughout the purification process.

### Analytical Size-Exclusion Chromatography.

WT and mutant DNMT3A proteins were subject to analytical size-exclusion chromatography on a Superose 6 Increase 10/300 GL column (GE Healthcare) at 4 °C. A total of 0.5 mL of protein at a concentration of ~0.6 mg mL^−1^ was loaded onto the column. Chromatography was performed at a flow rate of 0.5 mL min^−1^ in a buffer containing 20 mM HEPES-NaOH (pH 7.2), 250 mM NaCl, 5% (v/v) glycerol, and 5 mM DTT. Peak fractions were analyzed by SDS–PAGE.

### Mass Photometry.

Mass photometry measurements were performed on a Refeyn TwoMP instrument (Refeyn Ltd.). WT and mutant DNMT3A were diluted to a final concentration of 10 nM in a buffer containing 20 mM HEPES-NaOH (pH 7.2), 150 mM NaCl, and 5 mM DTT. Molecular weight calibration was performed using MassFerence P1 standard (Refeyn Ltd.), which contains four distinct species with molecular weights of 86 kDa, 172 kDa, 258 kDa, and 344 kDa. Data acquisition and analysis were carried out using AcquireMP (version 2025 1.2) and DiscoverMP (version 2025 1.2) software (Refeyn Ltd.), respectively.

### In Vitro DNA Methylation Assay.

A DNA fragment containing 4 × 177 Widom-601 sequence (59) was used as the methylation substrate. For the DNA methylation assays, 1 µg of DNA, 0.25 to 2 µM DNMT3A protein (residues 281 to 912 or DNMT3A^MTase^: 628 to 912), and 0.67 µM S-adenosyl-L-[methyl-^3^H] methionine (specific activity 89.2 Ci mmol^−1^; PerkinElmer) were incubated in a 20-µL buffer containing 50 mM Tris–HCl (pH 7.2), 0.05% (v/v) β-mercaptoethanol, 10% (v/v) glycerol, 0.01% (v/v) NP-40, and 200 µg mL^−1^ bovine serum albumin (BSA). Reactions were conducted in triplicate at 37 °C for 30 min. The reactions were terminated by the addition of 5 µL of 10 mM nonradioactive SAM. For quantification, 8 µL of each reaction mixture was spotted onto Amersham Hybond-N^+^ membranes (Cytiva) and air-dried. The membranes were washed twice with 0.2 M ammonium bicarbonate (pH 8.2), followed by sequential washes with Milli-Q water and ethanol. After drying, the membranes were transferred into scintillation vials containing 4 mL of ScintiVerse scintillation cocktail (Thermo Fisher Scientific). Tritium incorporation was measured using a Beckman LS6500 liquid scintillation counter.

### Cryo-EM Sample Preparation and Data Acquisition.

For cryoelectron microscopy (cryo-EM) analysis, freshly purified DNMT3A^WT^ homotetramer was subjected to size-exclusion chromatography on a Superose 6 10/300 column (Cytiva) equilibrated in 20 mM HEPES-NaOH (pH 7.2), 250 mM NaCl, 2.5% (v/v) glycerol, and 5 mM DTT. Fractions corresponding to the middle of the peak were collected and used immediately for grid preparation. An aliquot of 2.5 μL of DNMT3A^WT^ complex at a concentration of OD_280_ of 1 was applied to a Quantifoil holey carbon grid (Copper, R1.2/1.3, 300 mesh). The grids were glow-discharged for 1 min with air through PELCO easiGlow™ clean System (Ted Pella) before use. The grids were blotted and plunge-frozen in liquid ethane cooled by liquid nitrogen with a Vitrobot IV (Thermo Fisher) at 8 °C under 100% humidity. The frozen grids were stored in liquid nitrogen before use.

For the DNMT3A^R882H^ mutant, the untreated protein sample was highly heterogeneous. Therefore, chemical crosslinking was performed using the GraFix method (60). A total of 200 µL of freshly purified protein at 1.7 mg mL^−1^(~23.6 µM monomer concentration) was layered onto a 10 to 30% (w/v) linear glycerol gradient prepared in buffer containing 50 mM HEPES (pH 7.2) and 100 mM NaCl, with a glutaraldehyde gradient ranging from 0 to 0.03% (v/v). Ultracentrifugation was carried out at 40,000×*g* for 16 h at 4 °C using a Beckman SW41 rotor. Gradients were fractionated into 25 fractions (500 µL each). Fractions were analyzed by SDS–PAGE and negative-stain electron microscopy. Fractions exhibiting optimal homogeneity were pooled, dialyzed overnight against 20 mM Tris–HCl (pH 7.2), 250 mM NaCl, 2.5% (v/v) glycerol, and 1 mM DTT, and concentrated to OD_280_ of 0.5 (~4 µM monomer). Next, 2.5 μL of the protein sample was applied to a homemade Graphene Oxide coated Quantifoil holey carbon grid (Copper, R1.2/1.3, 300 mesh). The grids were frozen as described above.

High-resolution cryo-EM data were collected on a Titan Krios electron microscope operating at 300 keV, equipped with a post-GIF Gatan K3 Summit direct electron detector (counting mode) in National Cancer Institute Cryo-EM center (for DNMT3A^WT^) and NIH National Center for CryoEM Access and Training (NCCAT) (for DNMT3A^R882H^). For DNMT3A^WT^, movies were recorded at a nominal magnification of ×81,000 with a pixel of 1.09-Å and the defocus range of −1.0 to −2.0 μm. Each micrograph was recorded with 40 frames with a total dose rate of around 50 *e^−^*/Å^2^. For DNMT3A^R882H^, movies were recorded at a nominal magnification of ×81,000 with a pixel 1.083-Å and the defocus range of −0.6 to −2.0 μm. Each micrograph was recorded with 42 frames with a total dose rate of around 51.96 *e^−^*/Å^2^.

### Cryo-EM Data Processing and Model Building.

The cryo-EM data were processed using cryoSPARC (v4.7.1) (61). The movies were motion-corrected and dose-weighted using the patch-based motion correction module. The contrast transfer function (CTF) of the resulting images was then subject to patch-based estimation. For DNMT3A^WT^, a subset composed of 1,000 images was randomly selected and automatic blob particle picking was performed. After 2D classification and heterogenous refinement, 34,741 particles were selected as the template and subsequently template-based particle picking was performed. Totally, 3,196,889 particles were extracted from 5,055 images, with a down-scaled pixel size of 2.89-Å. After two rounds of clean-up by 2D classifications, those classes with identifiable secondary or tertiary structures, composed of 674,177 particles, were selected. Initial models were then generated by 3D ab initio reconstruction, followed by one round of heterogeneous refinement (K = 4) applying C1 symmetry. The class with well-defined density for DNMT3A homohexamer was selected and treated as “seed” particles and a round of seed-facilitated 3D classification (62) was performed. After removing duplicate particles, 804,797 high-quality particles were selected and subjected to one round of heterogeneous refinement (K = 4). Then, 313,002 high-quality particles were selected, re-extracted at a pixel size of 1.09-Å, and subjected to another round of heterogeneous refinement (K = 3). Finally, 197,622 particles with identified homohexamer features were selected for the nonuniform and local refinement applying C1 symmetry, achieving a global nominal resolution of approximately 3.66-Å. Due to the flexibility of two exterior subunits of DNMT3A homohexamer, the selected particles were then imported into RELION (v.5) for the DNMT3A homotetramer subtraction. The subtracted particles were further imported back and processed in cryoSPARC (v.4.7.1) and subjected to one round of heterogeneous refinement (K = 4). The class with well-defined density for DNMT3A homotetramer composed of 103,635 particles was selected for the final nonuniform refinement applying C1 symmetry to achieve the final overall resolution of 3.58-Å. The maps were sharpened in cryoSPARC for further model building. For visualization only, the experimental map was subject to processing by DeepEMhancer (63). The composite map of the DNMT3A homohexamer was generated by combining the sharpened consensus map of the homohexamer with the focused map of the homotetramer using Phenix (v1.21.2-5419).

For the data of DNMT3A^R882H^, the movies were motion-corrected and CTF estimated using patch-based module in cryoSPARC. Automatic particle picking was performed using a template-based method, in which the template was generated from 100 preprocessed images. Totally 5,142,136 particles were extracted from 25,240 images, with a down-scaled pixel size of 4.33 Å. After one round of clean-up by 2D classification, those classes with identifiable secondary or tertiary structures, composed of 283,105 particles, were selected and subsequently re-extracted at a pixel size of 1.35 Å. Initial models were then generated by 3D ab initio reconstruction followed by one round of heterogeneous refinement (K = 6). Two different classes were generated: 72,677 particles for state-I polymer and 32,187 particles for state-II polymer. After a final nonuniform and local refinement applying C2 symmetry to both classes in cryoSPARC, the resolution for state-I and state-II DNMT3A^R882H^ were determined at 4.19- and 5.04-Å, respectively, as given by the Fourier shell correlation criterion (FSC 0.143). The maps were sharpened in cryoSPARC for further model building.

For model building of DNMT3A homotetramer and hexamer, the reported structures of the DNMT3A^MTase^ homotetramer (PDB 8TDR) and DNMT3A2-DNMT3L (PDB 9PRW) were manually fit into the sharpened map in Chimera (v1.17.3) (64). The initial structural model was then subject to iterative model building using Coot (v0.9.8) (65) and real-space refinement using Phenix (v 1.21.2_5419) (66). For model building of DNMT3A^R882H^ state-I and state-II polymers, the reported structures of DNMT3A^MTase^ R882H/R676K homotetramer (PDB 8TE1) and DNMT3A2–DNMT3L complex (PDB 9PRW) were manually fit into the sharpened map and further subjected to iterative model building using Coot and real-space refinement using Phenix. For all the structures, a model-map Fourier shell correlation was calculated using the criterion of 0.5.

## Supplementary Material

Appendix 01 (PDF)

## Data Availability

Coordinates and structure factors for homo-oligomeric DNMT3A^WT^, homotetramer and homohexamer, and DNMT3A^R882H^, state-I and state-II polymers have been deposited in the Protein Data Bank under accession codes 11OH (67), 11OI (68), 11NZ (69), and 11OA (70), respectively. The cryo-EM density maps have been deposited to the EMDB under the accession numbers of EMD-75875 (consensus) (71) and EMD-75886 (composite) (72) for DNMT3A^WT^ homohexamer, EMD-75885 for DNMT3A^WT^ homotetramer (73), EMD-75876 for state-I DNMT3A^R882H^ polymer (74), and EMD-75877 for state-II DNMT3A^R882H^ polymer (75), respectively. All other data are included in the manuscript and/or *SI Appendix*.
