# Supplementary material for: Multidomain interaction governs the filamentous assembly of the dominant-negative DNMT3A R882H mutant
Source: Proc Natl Acad Sci U S A. 2026 Aug 25;123(35):e2609226123. doi: 10.1073/pnas.2609226123 (PMC13535186; doi:10.1073/pnas.2609226123)
Supplement: Supplementary file 1 [file pnas.2609226123.sapp.pdf]

Supplementary information for

Multidomain interaction governs the filamentous assembly of the dominant-negative DNMT3A R882H mutant

Jianbin Chen[1,#], Jiuwei Lu[1,#], Zhixu Long[1], Sol Yoon[1], Megan Fukunaga[1], Jikui Song[1,*]

[1]Department of Biochemistry, University of California, Riverside, CA 92521, USA

[#]These authors contributed to the project equally

*Correspondence: jikui.song@ucr.edu

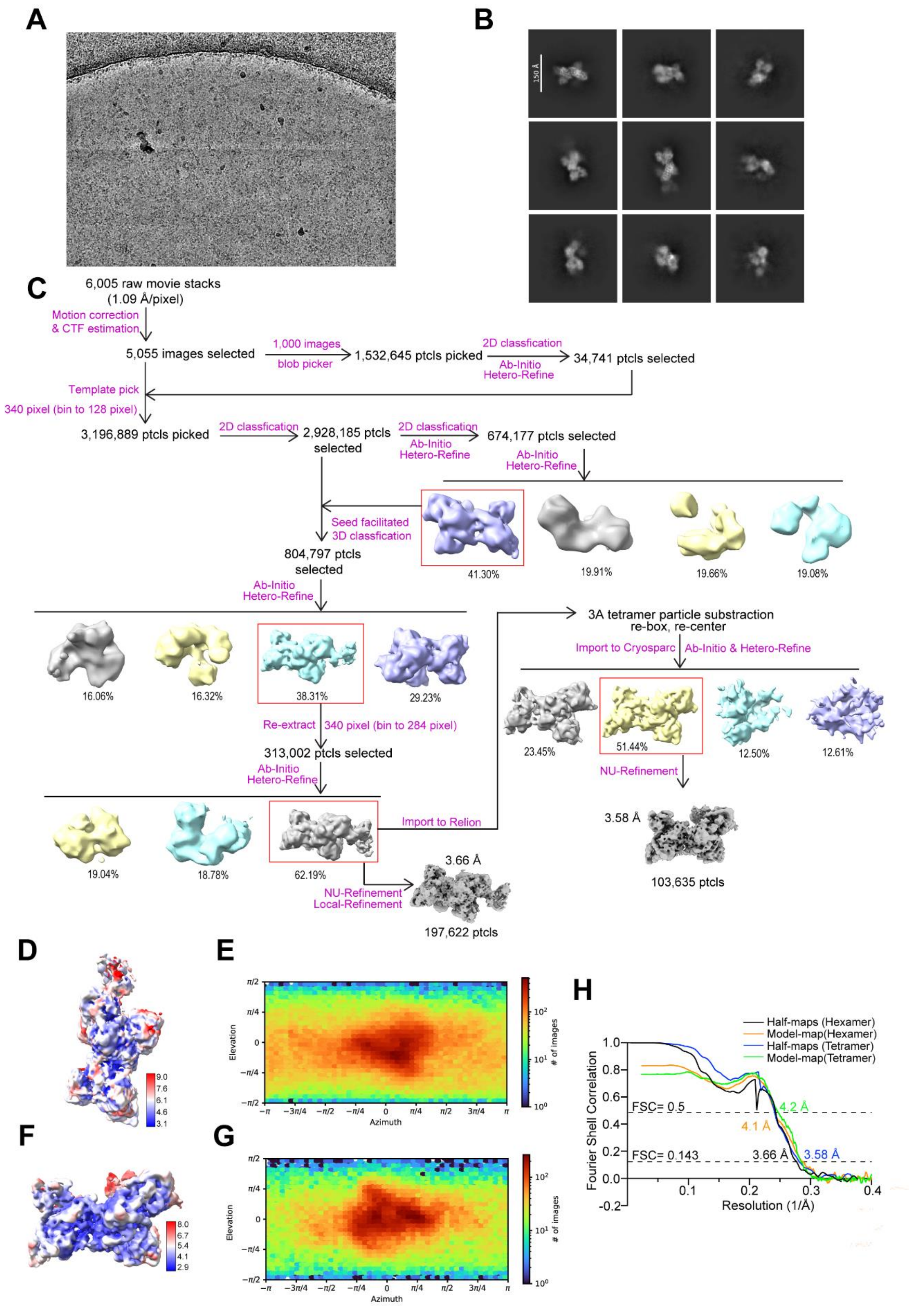

A
B
150 Å
C
6,005 raw movie stacks
(1.09 Å/pixel)
Motion correction
& CTF estimation
5,055 images selected
1,000 images
blob picker
1,532,645 ptcls picked
2D classfication
Ab-Initio
Hetero-Refine
34,741 ptcls selected
Template pick
340 pixel (bin to 128 pixel)
3,196,889 ptcls picked
2D classfication
2,928,185 ptcls
selected
2D classfication
Ab-Initio
Hetero-Refine
674,177 ptcls selected
Ab-Initio
Hetero-Refine
Seed facilitated
3D classfication
41.30%
19.91%
19.66%
19.08%
804,797 ptcls
selected
Ab-Initio
Hetero-Refine
16.06%
16.32%
38.31%
29.23%
Re-extract
340 pixel (bin to 284 pixel)
313,002 ptcls selected
Ab-Initio
Hetero-Refine
19.04%
18.78%
62.19%
Import to Relion
3.66 Å
NU-Refinement
Local-Refinement
197,622 ptcls
3A tetramer particle substraction
re-box, re-center
Import to Cryosparc
Ab-Initio & Hetero-Refine
23.45%
51.44%
12.50%
12.61%
NU-Refinement
3.58 Å
103,635 ptcls
D
9.0
7.6
6.1
4.6
3.1
E
Elevation
Azimuth
# of images
F
8.0
6.7
5.4
4.1
2.9
G
Elevation
Azimuth
# of images
H
Half-maps (Hexamer)
Model-map(Hexamer)
Half-maps (Tetramer)
Model-map(Tetramer)
Fourier Shell Correlation
FSC= 0.5
4.2 Å
4.1 Å
FSC= 0.143
3.66 Å
3.58 Å
Resolution (1/Å)

Figure S1. Cryo-EM structure determination of the DNMT3A^{WT} homo-oligomer. (A) A representative Cryo-EM micrograph of the DNMT3A^{WT} homo-oligomer. (B) Representative 2D classes of the DNMT3A^{WT} homo-oligomer from cryoSPARC. (C) Workflow for data processing, with the classes selected for next-step processing highlighted by red rectangle. (D-E) Local resolution (D) and angular distribution (E) of the final particles used for construction of DNMT3A^{WT} hexamer. (F-G) Local resolution (F) and angular distribution (G) of the final particles used for construction of DNMT3A^{WT} tetramer. (H) FSC curve for half maps and model-map for the structure of the DNMT3A^{WT} homo-oligomer.

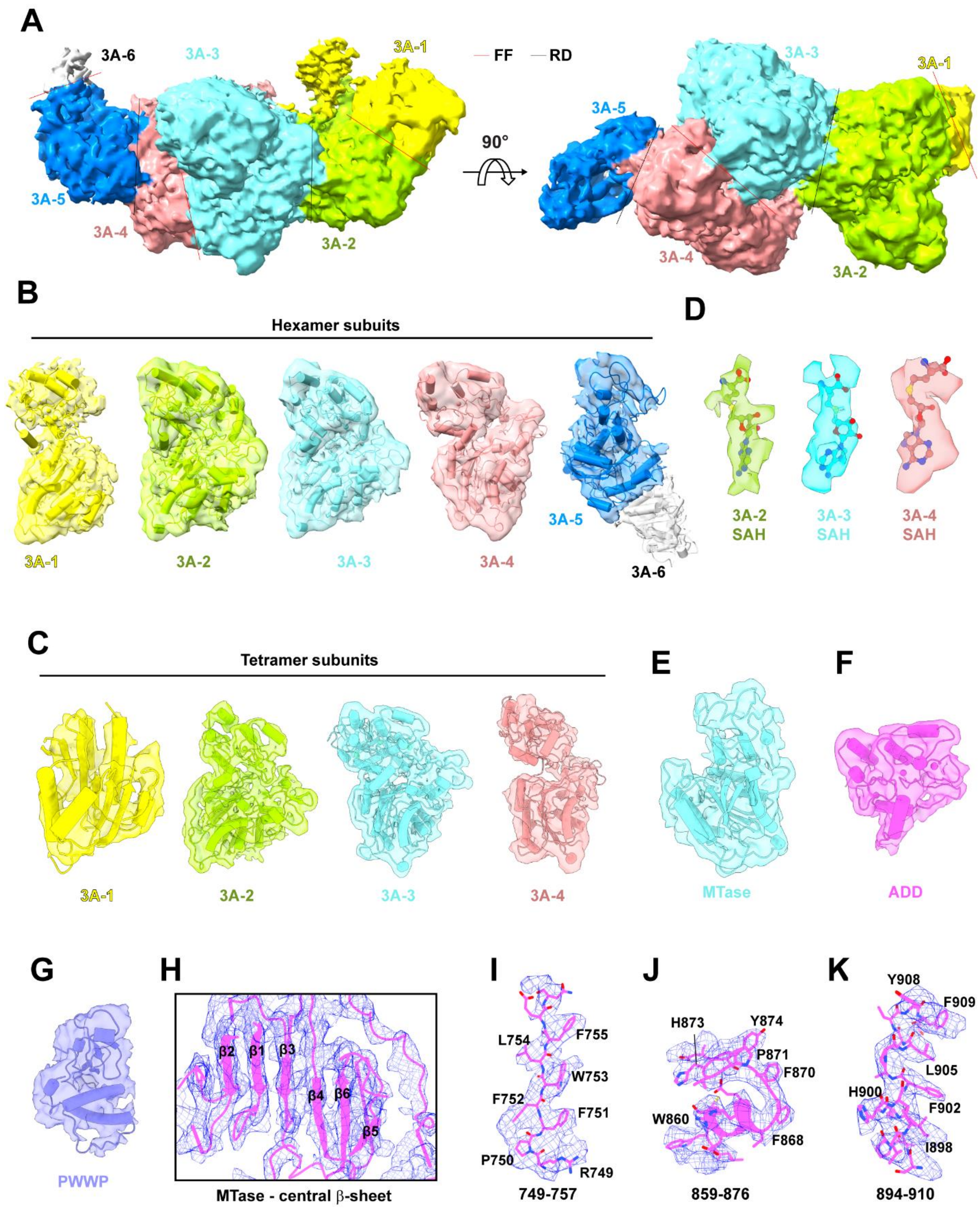

Figure S2. Cryo-EM density maps of the DNMT3A^{WT} homo-oligomer. (A) Two opposite views of the density map of the DNMT3A^{WT} hexamer, with individual subunits

color coded. The RD and FF interfaces are indicated by black and red lines, respectively. (B) Density maps and atomic models for the individual subunits of DNMT3A^{WT} hexamer. (C) Density maps and atomic models for the individual subunits of DNMT3A^{WT} tetramer. (D) Density maps for the SAH molecules bound to the 3A-2, 3A-3 and 3A-4 subunits of DNMT3A^{WT}. (E-G) Density maps and atomic models for the MTase (E), ADD (F) and PWWP (G) domains of the 3A-3 subunit of DNMT3A^{WT} tetramer. (H) Density map and atomic models for the central β-sheet of the 3A-3 MTase domain. (I-K) Density map and atomic models for representative residues with bulky side chains in the 3A-3 subunit.

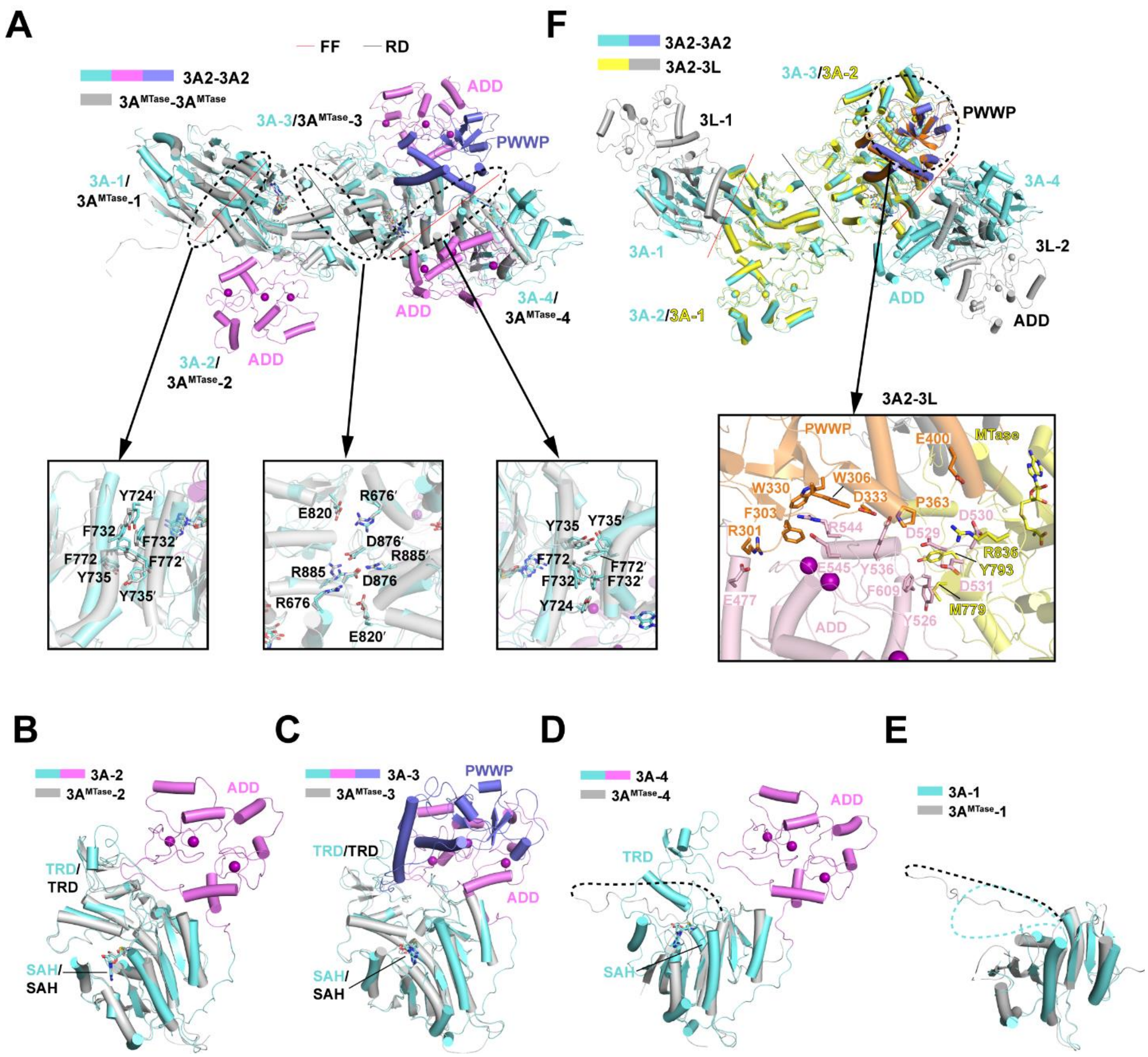

Figure S3. Structural comparison of DNMT3A^{WT} under various assembly states. (A) Structural overlay of DNMT3A^{WT} (3A2-3A2) and tetrameric DNMT3A MTase (3A^{MTase}) (PDB 8TDR), with residues involved in the FF and RD interfaces shown in the expanded views. (B) Structural overlay of the DNMT3A^{WT} 3A-2 subunit with the corresponding 3A^{MTase} subunit (3A^{MTase}-2) (PDB 8TDR). (C) Structural overlay of the DNMT3A^{WT} 3A-3 subunit with the corresponding 3A^{MTase} subunit (3A^{MTase}-3) (PDB 8TDR). (D) Structural overlay of the DNMT3A^{WT} 3A-4 subunit with the corresponding 3A^{MTase} subunit (3A^{MTase}-4) (PDB 8TDR). Note that the TRD is folded in the 3A-4 subunit of DNMT3A^{WT} but

disordered in $3A^{MTase}$-4 due to disruption of the RD interface. (E) Structural overlay of the DNMT3A^{WT} 3A-1 subunit with the corresponding $3A^{MTase}$ subunit ($3A^{MTase}$-1) (PDB 8TDR). (F) Structural overlay of DNMT3A^{WT} (3A2-3A2) and the DNMT3A2-DNMT3L complex (PDB 9PRW; 3A2-3L), with the residues involved in the trilateral PWWP-ADD-MTase interactions in DNMT3A2-DNMT3L shown in stick representation in the expanded view.

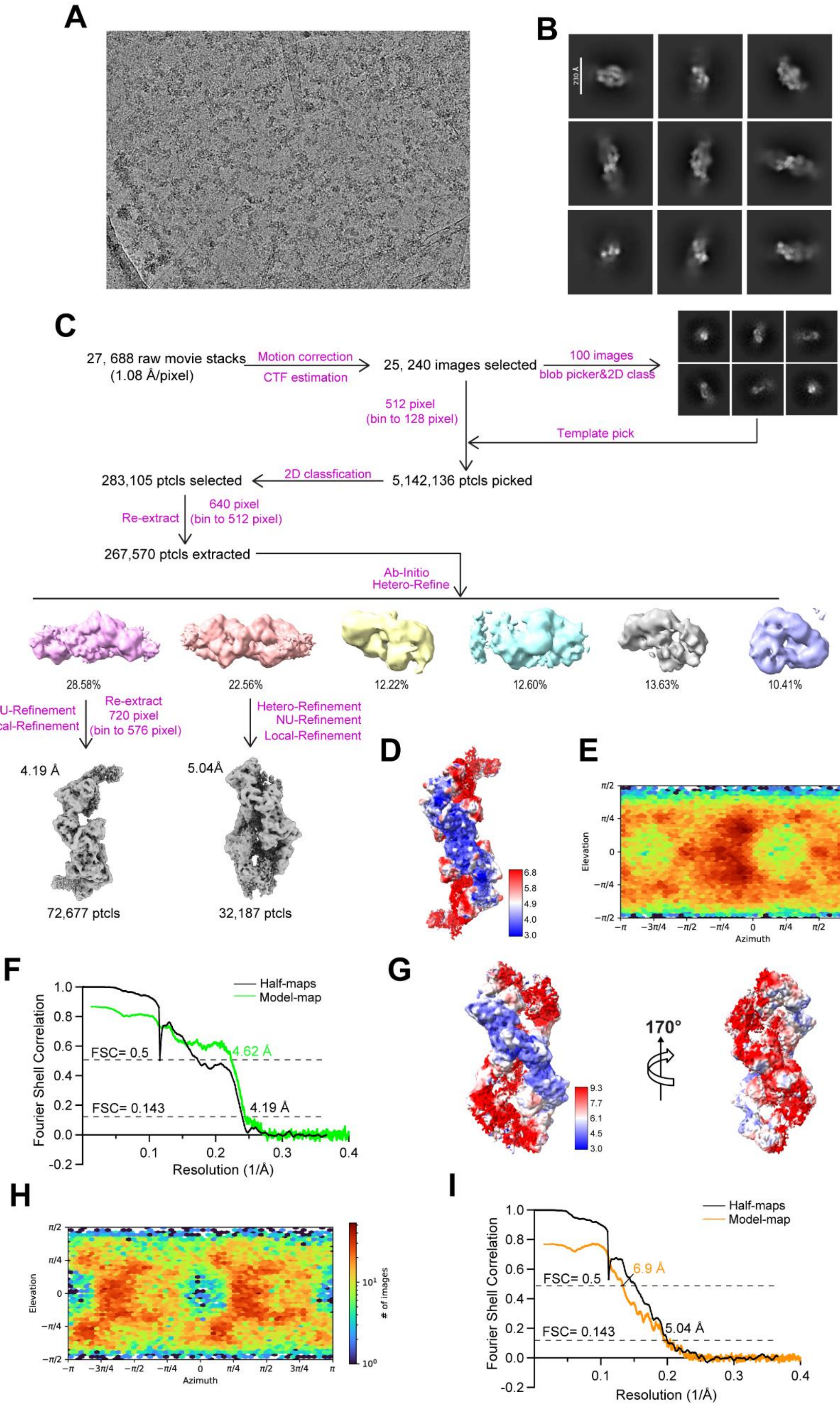

A
B
230 Å
C
27, 688 raw movie stacks
(1.08 Å/pixel)
Motion correction
CTF estimation
25, 240 images selected
100 images
blob picker&2D class
512 pixel
(bin to 128 pixel)
Template pick
283,105 ptcls selected
2D classfication
5,142,136 ptcls picked
Re-extract
640 pixel
(bin to 512 pixel)
267,570 ptcls extracted
Ab-Initio
Hetero-Refine
28.58%
22.56%
12.22%
12.60%
13.63%
10.41%
NU-Refinement
Local-Refinement
Re-extract
720 pixel
(bin to 576 pixel)
Hetero-Refinement
NU-Refinement
Local-Refinement
4.19 Å
5.04Å
72,677 ptcls
32,187 ptcls
D
E
Elevation
Azimuth
# of images
F
Half-maps
Model-map
Fourier Shell Correlation
FSC= 0.5
FSC= 0.143
4.62 Å
4.19 Å
Resolution (1/Å)
G
170°
H
Elevation
Azimuth
# of images
I
Half-maps
Model-map
Fourier Shell Correlation
FSC= 0.5
FSC= 0.143
6.9 Å
5.04 Å
Resolution (1/Å)

Figure S4. Cryo-EM structure determination of the DNMT3A^{R882H} homo-oligomer. (A) A representative Cryo-EM micrograph of the DNMT3A^{R882H} homo-oligomer. (B) Representative 2D classes of the DNMT3A^{R882H} homo-oligomer from cryoSPARC. (C) Workflow for data processing. (D-F) Local resolution (D) angular distribution (E) and FSC curve for half maps and model-map (F) for the state-I DNMT3A^{R882H} polymer. (G-I) Local resolution (G), angular distribution (H) and FSC curve for half maps and model-map (I) for the state-II DNMT3A^{R882H} polymer.

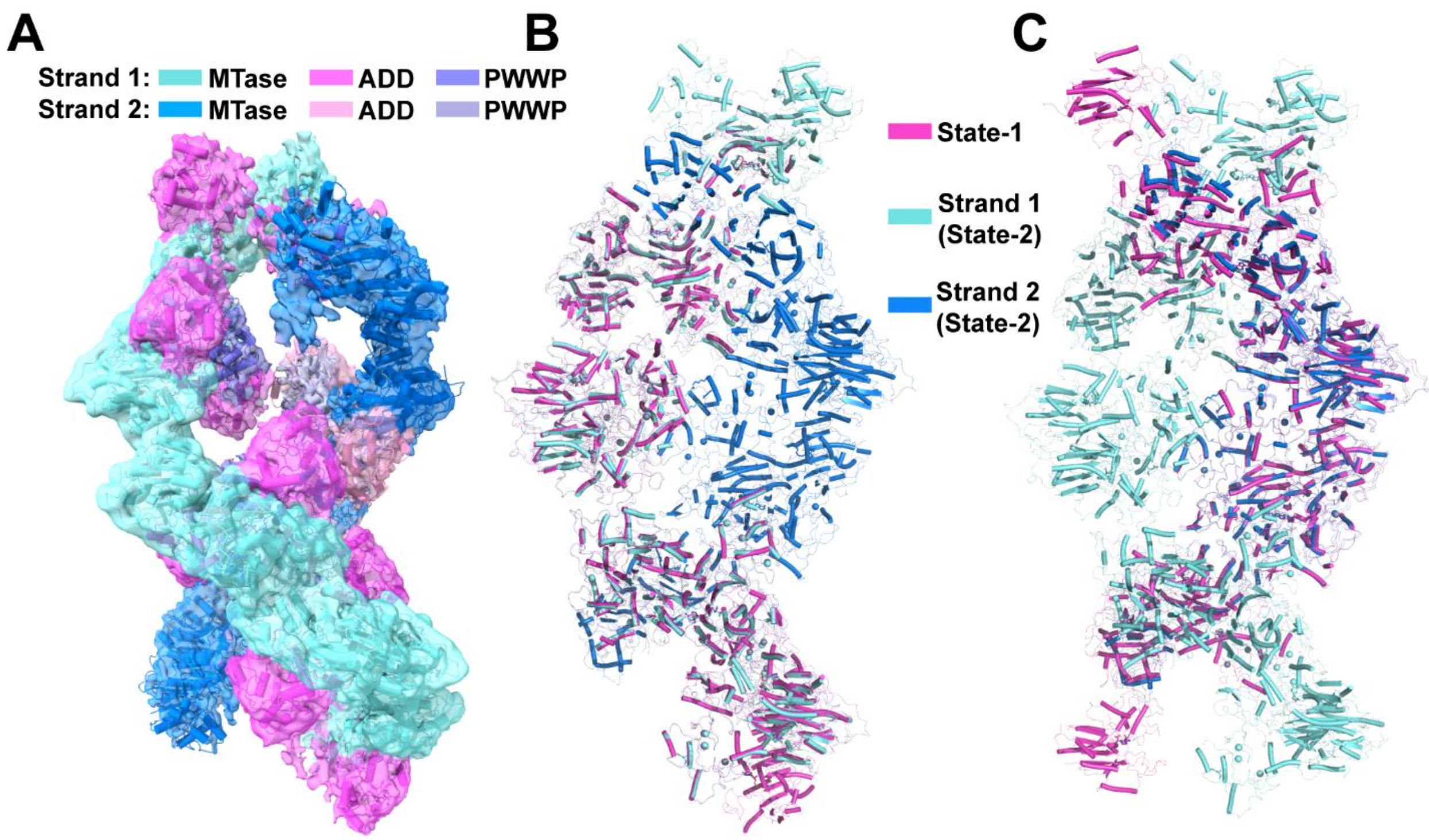

Figure S5. Structural analysis of the DNMT3A^{R882H} polymer. (A) Density map of the state-II DNMT3A^{R882H} polymer at contour level of 0.13, overlaid with the atomic model. (B-C) Structural overlay of the state-I DNMT3A^{R882H} polymer with the strand-1 (B) and strand-2 (C) of the state-II DNMT3A^{R882H} polymer.

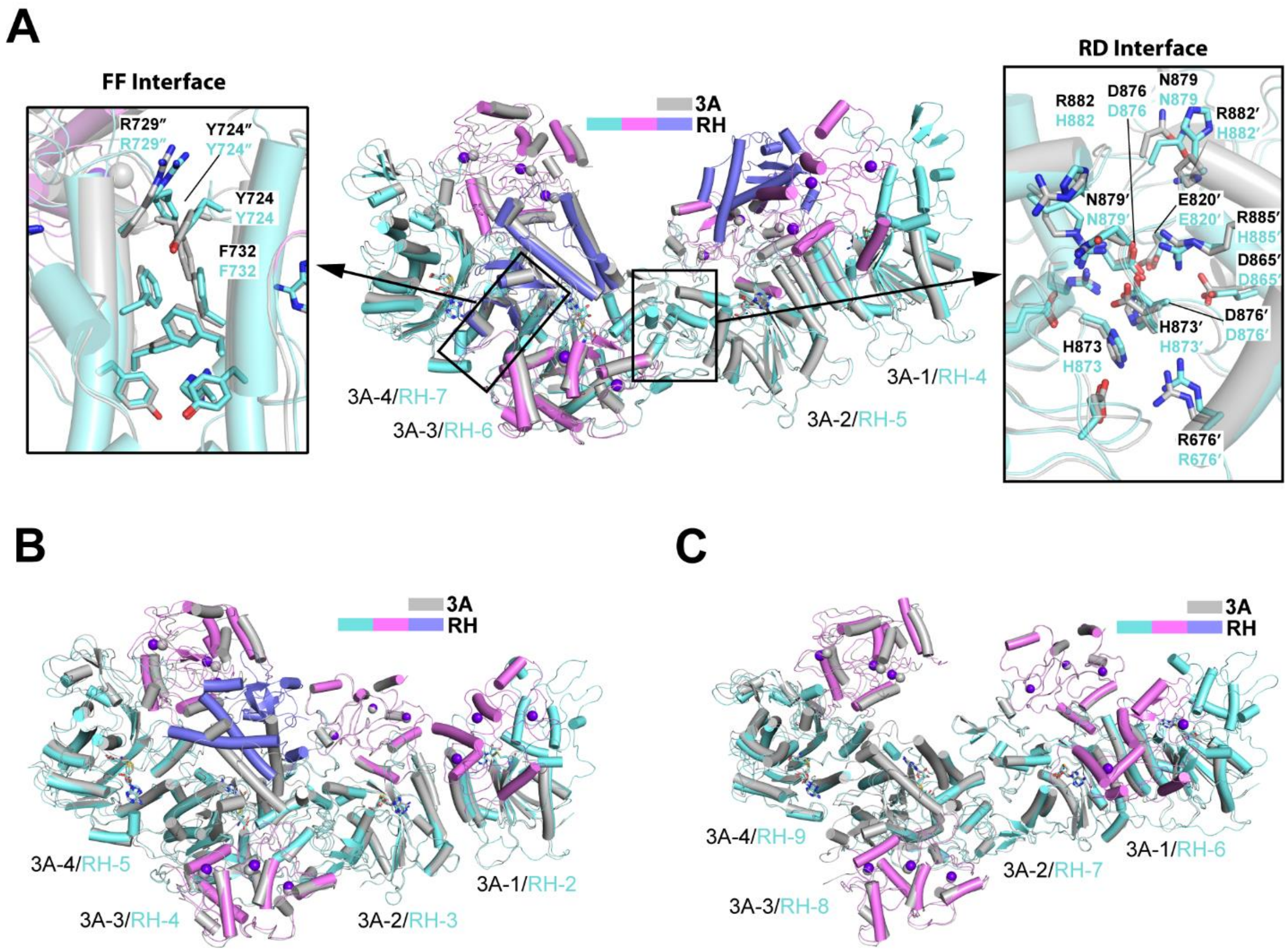

Figure S6. Structural comparison of DNMT3A^{WT} tetramer and state-I DNMT3A^{R882H}. (A) Structural overlay of DNMT3A^{WT} tetramer and the RH-4/RH-5/RH-6/RH-7 segment of state-I DNMT3A^{R882H}, with residues involved in the FF interface and the RD interfaces shown in the expanded views. (B-C) Structural overlay of DNMT3A^{WT} tetramer and the RH-2/RH-3/RH-4/RH-5 (B) and RH-6/RH-7/RH-8/RH-9 (C) segments of state-I DNMT3A^{R882H}.

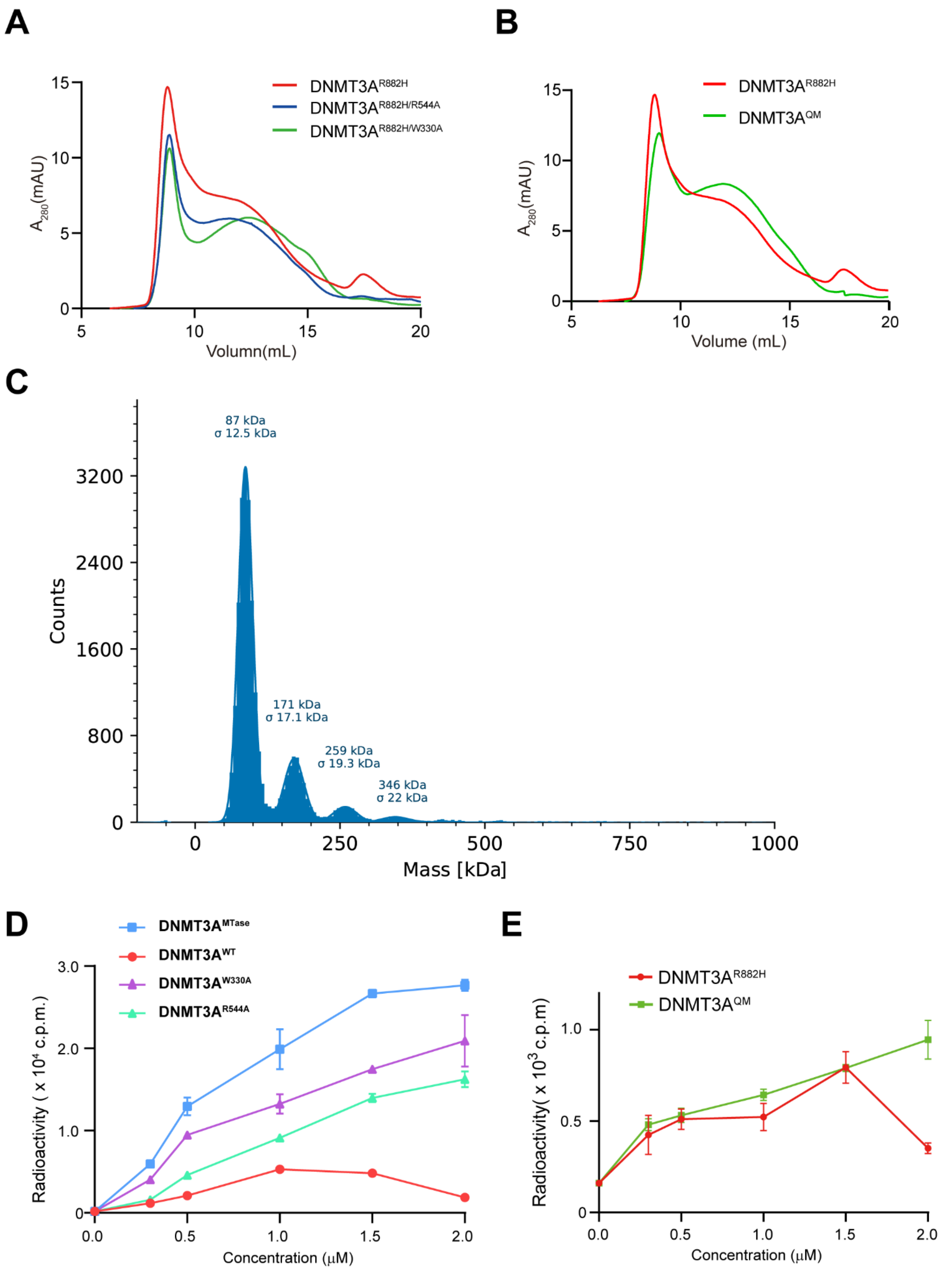
A
DNMT3AR882H
DNMT3AR882H/R544A
DNMT3AR882H/W330A
A280(mAU)
Volumn(mL)
B
DNMT3AR882H
DNMT3AQM
A280(mAU)
Volume (mL)
C
87 kDa
σ 12.5 kDa
171 kDa
σ 17.1 kDa
259 kDa
σ 19.3 kDa
346 kDa
σ 22 kDa
Counts
Mass [kDa]
D
DNMT3AMTase
DNMT3AWT
DNMT3AW330A
DNMT3AR544A
Radioactivity (x 10⁴ c.p.m.)
Concentration (μM)
E
DNMT3AR882H
DNMT3AQM
Radioactivity(x 10³ c.p.m)
Concentration (μM)

Figure S7. Biochemical analysis of DNMT3A polymerization. (A) Size-exclusion chromatography analysis of the PWWP-ADD-MTase fragment of DNMT3A^{R882H}, DNMT3A$^{R882H/W330A}$ and DNMT3A$^{R882H/R544A}$. (B) Size-exclusion chromatography analysis of the PWWP-ADD-MTase fragment of DNMT3A^{R882H} and DNMT3A$^{R882H/Q573G/A574G/A575G}$ (DNMT3A^{QM}). (C) Mass photometry profile of the MassFerence™ P1 Calibrant used for molecular weight calibration in this study. (D) Protein concentration-dependent DNA methylation activity of DNMT3A PWWP-ADD-MTase, WT or mutant, as well as DNMT3A^{MTase} (n = 3 biological replicates). (E) Protein concentration-dependent DNA methylation activity of DNMT3A^{R882H} and DNMT3A^{QM} in the context of the PWWP-ADD-MTase fragment (n = 3 biological replicates).

Table S1. Cryo-EM data collection, structure refinement and validation statistics

	DNMT3A^WT		DNMT3A^R882H	
**State**	**Hexamer**	**Tetramer**	**State-I (single-stranded filament)**	**State-II (double-stranded filament)**
**Codes**	EMD-75875 (Consensus), EMD-75886 (Composite) PDB 11OI	(EMD-75885, PDB 11OH)	(EMD-75876, PDB 11NZ)	(EMD-75877, PDB 11OA)
**Data collection and processing**				
Microscope	TFS Krios		TFS Krios	
Camera	Gatan K3 (6K × 4K)		Gatan K3 (6K × 4K)	
Magnification	81,000		81,000	
Voltage (kV)	300		300	
Defocus range (µm)	-1.0 ~ -2.0		-0.6 ~ -2.0	
Exposure time (s)	2.6		2.0	
Dose rate(e^-/Å^2/s)	19.23		25	
Number of frames	40		42	
Pixel size (Å)	1.09		1.083	
Micrographs (no.)	6,005		27,688	
Initial particles (no.)	3,196,889		5,142,136	
Symmetry imposed	*C1*	*C1*	*C2*	*C2*
Final particles (no.)	197,622	103,635	72,677	32,187
Map resolution (Å)	3.66	3.58	4.19	5.04
FSC threshold	0.143	0.143	0.143	0.143
**Refinement**				
Initial model used	9PRW, 8TDR	9PRW, 8TDR	9PRW, 8TE1	9PRW, 8TE1
Model resolution (Å)	4.1	4.2	4.62	6.9
FSC threshold	0.5	0.5	0.5	0.5
Map sharpening *B* factor (Å^2)	-50	-63.88	-50	-100
Model composition				
Non-hydrogen atoms	17,511	12,658	33,534	57,257
Protein residues	2,314	1,364	4,501	7,577
Ion (zinc)	12	9	24	42
SAH	4	3	10	16
B factors (Å^2)				
Protein	255.77	214.93	325.62	602.09
Ion (zinc)	346.56	301.96	509.51	811.99
SAH	199.8	171.17	226.08	576.29
R.m.s. deviations				
Bond lengths (Å)	0.006	0.003	0.005	0.004
Bond angles (°)	0.658	0.511	0.989	0.96
**Validation**				
MolProbity score	2.03	1.89	1.91	1.77
Clashscore	11.74	8.53	9.02	7.76
Poor rotamers (%)	1.18	1.2	0.16	0.13
Ramachandran plot				
Favored (%)	94.2	94.56	93.53	94.96
Allowed (%)	5.8	5.44	6.47	5.04
Disallowed (%)	0	0	0	0
